# Yellow Fever in Africa: Estimating the Burden of Disease and Impact of Mass Vaccination from Outbreak and Serological Data

**DOI:** 10.1371/journal.pmed.1001638

**Published:** 2014-05-06

**Authors:** Tini Garske, Maria D. Van Kerkhove, Sergio Yactayo, Olivier Ronveaux, Rosamund F. Lewis, J. Erin Staples, William Perea, Neil M. Ferguson, for the Yellow Fever Expert Committee

**Affiliations:** 1 MRC Centre for Outbreak Analysis, Department of Infectious Disease Epidemiology, Imperial College London, United Kingdom; 2 World Health Organization, Geneva, Switzerland; 3 Immunization and Vaccine Development, World Health Organization, Ouagadougou, Burkina Faso; 4 Ottawa Public Health, Ottawa, Ontario, Canada; 5 Arboviral Disease Branch, Centers for Disease Control and Prevention, Fort Collins, Colorado, United States of America; University of Oxford, United Kingdom

## Abstract

**Background:**

Yellow fever is a vector-borne disease affecting humans and non-human primates in tropical areas of Africa and South America. While eradication is not feasible due to the wildlife reservoir, large scale vaccination activities in Africa during the 1940s to 1960s reduced yellow fever incidence for several decades. However, after a period of low vaccination coverage, yellow fever has resurged in the continent. Since 2006 there has been substantial funding for large preventive mass vaccination campaigns in the most affected countries in Africa to curb the rising burden of disease and control future outbreaks. Contemporary estimates of the yellow fever disease burden are lacking, and the present study aimed to update the previous estimates on the basis of more recent yellow fever occurrence data and improved estimation methods.

**Methods and Findings:**

Generalised linear regression models were fitted to a dataset of the locations of yellow fever outbreaks within the last 25 years to estimate the probability of outbreak reports across the endemic zone. Environmental variables and indicators for the surveillance quality in the affected countries were used as covariates. By comparing probabilities of outbreak reports estimated in the regression with the force of infection estimated for a limited set of locations for which serological surveys were available, the detection probability per case and the force of infection were estimated across the endemic zone.

The yellow fever burden in Africa was estimated for the year 2013 as 130,000 (95% CI 51,000–380,000) cases with fever and jaundice or haemorrhage including 78,000 (95% CI 19,000–180,000) deaths, taking into account the current level of vaccination coverage. The impact of the recent mass vaccination campaigns was assessed by evaluating the difference between the estimates obtained for the current vaccination coverage and for a hypothetical scenario excluding these vaccination campaigns. Vaccination campaigns were estimated to have reduced the number of cases and deaths by 27% (95% CI 22%–31%) across the region, achieving up to an 82% reduction in countries targeted by these campaigns. A limitation of our study is the high level of uncertainty in our estimates arising from the sparseness of data available from both surveillance and serological surveys.

**Conclusions:**

With the estimation method presented here, spatial estimates of transmission intensity can be combined with vaccination coverage levels to evaluate the impact of past or proposed vaccination campaigns, thereby helping to allocate resources efficiently for yellow fever control. This method has been used by the Global Alliance for Vaccines and Immunization (GAVI Alliance) to estimate the potential impact of future vaccination campaigns.

*Please see later in the article for the Editors' Summary*

## Abstract 2

Neil Ferguson and colleagues estimate the disease burden of yellow fever in Africa, as well as the impact of mass vaccination campaigns.

*Please see later in the article for the Editors' Summary*

## Editors' Summary

**Background:**

Yellow fever is a flavivirus infection that is transmitted to people and to non-human primates through the bites of infected mosquitoes. This serious viral disease affects people living in and visiting tropical regions of Africa and Central and South America. In rural areas next to forests, the virus typically causes sporadic cases or even small-scale epidemics (outbreaks) but, if it is introduced into urban areas, it can cause large explosive epidemics that are hard to control. Although many people who contract yellow fever do not develop any symptoms, some have mild flu-like symptoms, and others develop a high fever with jaundice (yellowing of the skin and eyes) or hemorrhaging (bleeding) from the mouth, nose, eyes, or stomach. Half of patients who develop these severe symptoms die. Because of this wide spectrum of symptoms, which overlap with those of other tropical diseases, it is hard to diagnose yellow fever from symptoms alone. However, serological tests that detect antibodies to the virus in the blood can help in diagnosis. There is no specific antiviral treatment for yellow fever but its symptoms can be treated.

**Why Was This Study Done?:**

Eradication of yellow fever is not feasible because of the wildlife reservoir for the virus but there is a safe, affordable, and highly effective vaccine against the disease. Large-scale vaccination efforts during the 1940s, 1950s, and 1960s reduced the yellow fever burden for several decades but, after a period of low vaccination coverage, the number of cases rebounded. In 2005, the Yellow Fever Initiative—a collaboration between the World Health Organization (WHO) and the United Nations Children Fund supported by the Global Alliance for Vaccines and Immunization (GAVI Alliance)—was launched to create a vaccine stockpile for use in epidemics and to implement preventive mass vaccination campaigns in the 12 most affected countries in West Africa. Campaigns have now been implemented in all these countries except Nigeria. However, without an estimate of the current yellow fever burden, it is hard to determine the impact of these campaigns. Here, the researchers use recent yellow fever occurrence data, serological survey data, and improved estimation methods to update estimates of the yellow fever burden and to determine the impact of mass vaccination on this burden.

**What Did the Researchers Do and Find?:**

The researchers developed a generalized linear statistical model and used data on the locations where yellow fever was reported between 1987 and 2011 in Africa, force of infection estimates for a limited set of locations where serological surveys were available (the force of infection is the rate at which susceptible individuals acquire a disease), data on vaccination coverage, and demographic and environmental data for their calculations. They estimate that about 130,000 yellow fever cases with fever and jaundice or hemorrhage occurred in Africa in 2013 and that about 78,000 people died from the disease. By evaluating the difference between this estimate, which takes into account the current vaccination coverage, and a hypothetical scenario that excluded the mass vaccination campaigns, the researchers estimate that these campaigns have reduced the burden of disease by 27% across Africa and by up to 82% in the countries targeted by the campaigns (an overall reduction of 57% in the 12 targeted countries).

**What Do These Findings Mean?:**

These findings provide a contemporary estimate of the burden of yellow fever in Africa. This estimate is broadly similar to the historic estimate of 200,000 cases and 30,000 deaths annually, which was based on serological survey data obtained from children in Nigeria between 1945 and 1971. Notably, both disease burden estimates are several hundred-fold higher than the average number of yellow fever cases reported annually to WHO, which reflects the difficulties associated with the diagnosis of yellow fever. Importantly, these findings also provide an estimate of the impact of recent mass vaccination campaigns. All these findings have a high level of uncertainty, however, because of the lack of data from both surveillance and serological surveys. Other assumptions incorporated in the researchers' model may also affect the accuracy of these findings. Nevertheless, the framework for burden estimation developed here provides essential new information about the yellow fever burden and the impact of vaccination campaigns and should help the partners of the Yellow Fever Initiative estimate the potential impact of future vaccination campaigns and ensure the efficient allocation of resources for yellow fever control.

**Additional Information:**

Please access these websites via the online version of this summary at http://dx.doi.org/10.1371/journal.pmed.1001638.

- The World Health Organization provides detailed information about yellow fever (in several languages), including photo stories about vaccination campaigns in the Sudan and Mali; it also provides information about the Yellow Fever Initiative (in English and French)
- The GAVI Alliance website includes detailed of its support for yellow fever vaccination
- The US Centers for Disease Control and Prevention provides information about yellow fever for the public, travelers, and health care providers
- The UK National Health Service Choices website also has information about yellow fever
- Wikipedia has a page on yellow fever that includes information about the history of the disease (note that Wikipedia is a free online encyclopedia that anyone can edit; available in several languages)

## Introduction

Yellow fever is a flavivirus infection that is transmitted primarily by mosquitoes of the species *Aedes* ssp. and *Haemagogus* spp., with humans and non-human primates being the main vertebrate hosts. It is endemic in tropical areas of Africa and Central and South America. The clinical course of infection in humans shows a wide spectrum of severity including asymptomatic infection, mild illness with flu-like symptoms, and severe disease including fever with jaundice or haemorrhage and death.

Several different transmission cycles have been defined, depending on which host and vector species are involved in transmission: in the sylvatic cycle, tree-dwelling mosquitoes of *Aedes* spp. (Africa) or *Haemagogus* spp. (Americas) transmit the virus to non-human primates. In this cycle, spill-over infection of humans occurs when they encroach on this jungle habitat. Conversely, in the urban transmission cycle, humans are the main hosts with transmission occurring via domestic mosquito species. The typical urban vector is *Aedes aegyptii*, which also serves as the main vector for dengue virus transmission. If yellow fever is introduced into urban areas, large explosive outbreaks can occur, which can be difficult to control. In Africa, there is also an intermediate transmission cycle that occurs in rural areas typically at the edges of forests with humans as well as non-human primates affected, and transmission driven by domestic and semi-domestic mosquito species [1],[2].

While eradication of yellow fever is not feasible due to the sylvatic reservoir, a high level of control is achievable owing to the availability of an efficacious and safe vaccine that confers long-lasting immunity from a single dose. Visas for many countries worldwide require proof of previous vaccination against yellow fever, particularly if travelers come from or have visited yellow fever endemic areas, in order to prevent the importation of the disease.

Quantifying the burden of disease caused by yellow fever is made challenging by the wide spectrum of clinical severity, with non-specific symptoms in the majority of infections making diagnosis difficult. In addition, there are considerable limitations in the surveillance and health care systems across much of the affected regions. However, it is clear that yellow fever is substantially underreported [3],[4]. Previous estimates from the early 1990s placed the burden of disease at 200,000 cases and 30,000 deaths annually [5],[6]. These estimates relied heavily on data from serological surveys performed in children in Nigeria between 1945 and 1971 [7]. These data still form the basis of more recent efforts to quantify disease burden or the cost-effectiveness of vaccines [8],[9]. More recent approaches to quantify yellow fever circulation have focused on producing risk maps [10]–[12], frequently employing regression techniques similar to the approach we adopt [10],[12], or relying on expert advice regarding local yellow fever distribution [11],[12]. However, there are no recent estimates of the yellow fever burden that take into account more recent surveillance and serological data and that account for vaccination coverage.

In 2005, the Yellow Fever Initiative was launched as a collaboration between WHO and the United Nations Children's Fund (UNICEF) with support from the Global Alliance for Vaccines and Immunization (GAVI Alliance). The aim was to secure the precarious yellow fever vaccine supply by creating a vaccine stockpile to be used in outbreak response campaigns as well as to increase the vaccination coverage in the most affected areas by implementation of large preventive mass vaccination campaigns in 12 of the most affected countries in West Africa. Between 2006 and 2012, these campaigns have been implemented in all of these countries apart from Nigeria because of larger than anticipated vaccine needs and limited vaccine supplies. In the same time frame, the Central African Republic, though not covered under the Yellow Fever Initiative, also performed mass vaccination campaigns with support from the GAVI Alliance.

During the October 2011 meeting of the advisory committee on Quantitative Immunization and Vaccine Related Research ([QUIVER], currently named Immunization and Vaccines related Implementation Research [IVIR]), the Advisory Committee recommended that WHO establish a working group to generate updated yellow fever disease burden estimates for Africa. This paper reports the results of this activity, presenting new estimates of the disease burden caused by yellow fever in Africa and the impact of preventive vaccination campaigns carried out under the Yellow Fever Initiative. The estimates are derived from a coherent model framework that integrates all available data including incidence, serology, and vaccination coverage.

## Methods

### Overview

We fitted a generalised linear model to the locations where yellow fever was reported in the 25-year period between 1987 and 2011. This model estimated, for each location, the probability of at least one yellow fever report over the observation period. The number of infections required to give rise to these probabilities of occurrence was then estimated by taking into account the probability of detection of yellow fever cases in each country. Estimated numbers of infections were converted to estimates of the force of infection using data on the population size, age distribution, and age-specific vaccination coverage in the observation period. Again using demographic and vaccination coverage data, the burden in terms of the number of infections, severe cases presenting with fever and jaundice or haemorrhage, or deaths can then be obtained from the estimates of the force of infection for each location for any year in the past or future, given assumptions on population growth and size of future vaccination campaigns.

### Datasets

The model was fitted at a spatial resolution of the first sub-national administrative unit (which in many countries is called “province”; this is the terminology adopted throughout this manuscript), so all datasets were resolved or aggregated to this level as appropriate.

#### Yellow fever occurrence

A database of the locations of reported outbreaks between 1987 and 2011 was compiled from various sources including the Weekly Epidemiological Record (WER) [13], the WHO disease outbreak news (DON) [14], an internal WHO database of outbreaks between 1980 and 2007, and the published literature. Locations were resolved to the province level, and data were recorded for each outbreak on the year of occurrence, size, and control measures implemented. Outbreak reports that could not be located at the province level were excluded.

In 2005, the African Regional Office of WHO established a yellow fever surveillance database (YFSD) of reports of suspected yellow fever cases (based primarily on a case definition of fever with jaundice) across 21 countries in West and central Africa. Data fields recorded for each case included age, gender, location, disease onset date, and the status of laboratory confirmation. The locations of all lab-confirmed cases between 2005 and 2011, resolved to the province level, were combined with the outbreaks dataset to generate an overall dataset of the areas of yellow fever occurrence, recording for each province whether or not there had been at least one yellow fever outbreak or case report in the period from 1987 to 2011.

Due to the very low proportion of suspected cases actually being attributed to yellow fever in the YFSD, the majority of cases reported likely had other causes (for instance viral hepatitis). Hence the national incidence of suspected cases is best interpreted as a measure of the effort put into yellow fever surveillance rather than a measure of yellow fever incidence itself. The incidence of suspected cases was aggregated at the country level and divided by the national population to be used as a covariate in the regression models fitted throughout.

#### Disease severity

The proportion of infections presenting as severe cases and the proportion of severe cases resulting in death varies substantially between settings, depending on previous exposure to other flaviviruses, but also factors such as clinical care and importantly detection bias due to surveillance coverage or case definitions used [1],[15]–[20]. Recent work by Johansson and colleagues [21] has estimated the proportion of infections that are asymptomatic, cause mild symptoms (excluding jaundice and haemorrhage), or severe symptoms (including jaundice, haemorrhage, or death), as well as the proportion of severe cases leading to death. We use these estimates of 13% (95% CI 5%–28%) of infections presenting as severe cases, and 46% (95% CI 31%–60%) of severe cases resulting in death to estimate the number of severe cases and deaths from the number of infections estimated by our model.

#### Vaccination coverage

No comprehensive dataset of yellow fever vaccination coverage in the endemic area in Africa was available, so vaccination coverage was estimated using data on (i) large-scale mass vaccination activities in French West Africa during the 1940s to 1960s [22]; (ii) outbreak response campaigns since 1970, as reported in outbreak reports in the WER or DONs [13],[14]; (iii) routine infant yellow fever vaccination occurring as part of the Enhanced Programme for Immunization (EPI) [23]; and (iv) mass vaccination campaigns in 11 West African countries under the Yellow Fever Initiative and the Central African Republic from 2006 to 2012 [24],[25].

Information on yellow fever vaccination was compiled into a dataset of age-specific vaccination coverage at the second sub-national administrative level (district), taking into account the location and extent of each campaign as well as the demographics of the targeted populations. This dataset allowed the achieved coverage to be tracked through time for each birth cohort in each district.

The available information on vaccination activities varied greatly from country to country, sometimes specifying the coverage achieved in a certain area, sometimes the number of doses administered during a vaccination campaign, and sometimes both. If the area targeted by a campaign was well defined geographically we used information on the vaccination coverage achieved by that campaign in preference to the number of doses administered in order to avoid uncertainty in population size affecting our estimates. If no information on the coverage achieved was available or the target population was not sufficiently well defined, we calculated vaccination coverage as the number of doses administered divided by the population size, assuming that individuals from all targeted age groups had an equal chance of receiving the vaccine, and that vaccination was performed irrespective of previous vaccination or disease history.

From the vaccination coverage achieved in individual vaccination campaigns the coverage at the population level over time was obtained by tracking vaccination coverage in each birth cohort. In compiling the vaccination coverage dataset, population movements were ignored, and 100% vaccine efficacy was assumed, with lifelong protection. The last two assumptions are supported by data showing that 99% of individuals seroconvert within 30 days of vaccination [1],[26], and neutralising antibodies have been measured 35 years post vaccination [26]–[28].

In estimating the impact of potential future vaccination campaigns we assumed that no further outbreak response vaccination campaigns would be undertaken and that the country-specific coverage in the infant immunization campaigns would be held constant at the levels estimated for 2011 (see Table S1) [23].

#### Serological surveys

Serological surveys have been used historically to assess overall levels of transmission. All literature on yellow fever serologic surveys conducted in Africa and published since 1980 were reviewed and the results collated [21]. For the analysis of transmission intensity, only surveys that had samples tested for yellow fever virus specific neutralising antibodies and were not part of an outbreak investigation were considered [29]–[34], as surveys conducted in outbreak situations are typically not representative. Even if random population samples are obtained in an outbreak-associated survey, serology would be expected to yield information on the attack rate for that specific outbreak rather than the average force of infection over a longer time period.

#### Demographic data

Demographic data on population size and age distribution at a sub-national level were used to interpret the data on vaccination campaigns as well as for estimating the burden. We used UN World population prospects (WPPs) [35] estimates of the population size by country in 5-year age bands for each year between 1950 and 2100. In order to achieve a higher spatial resolution of the population distribution, these estimates were combined with the LandScan 2007 dataset [36],[37], which gave estimates for the year 2007 of the total population on a grid of resolution of 1/120 degree latitude and longitude, which is approximately 1 km at the equator. By allocating each grid point to the second sub-national administrative unit (which in many countries is the district), the proportion of each country's population living in any particular district was estimated. In the absence of more detailed datasets, it was assumed that the age distributions were homogeneous within each country, neglecting local differences, for instance between rural and urban areas. We furthermore assumed that population growth was homogeneous within a country, and that the population proportions for each district obtained from the LandScan 2007 dataset were applicable to all other years. Thus we did not capture trends in urbanisation or other shifts in the relative population sizes of different districts over time.

We disaggregated the 5-year age bands of the UN WPP dataset into annual birth cohorts using the method described in Text S1.

Population based variables for the regression model included the total population for each province, the logarithm of the population size and the proportion of the population living in urban areas (defined as LandScan 2007 dataset pixels with a population density of ≥386 people per sq km [38]).

#### Environmental data

Environmental datasets on rainfall [39], day- and night-time air temperatures [40], land cover classifications [41],[42], the enhanced vegetation index (EVI), the middle infrared reflectance (MIR) [43], longitude, latitude, and altitude [44],[45] were used as potential covariates in the generalised linear model. These data were available as gridded datasets of various spatial resolutions between about 1 km and 10 km, and were aggregated to province level by calculating the mean value for each variable, weighted by the population size attributed to each grid cell in order to obtain values representative of the conditions where human populations are concentrated.

For the land cover classification, the proportion of pixels (weighted by population size) for each category was aggregated for each province to obtain scalar variables. In the endemic zone, some of the 17 defined land cover classes occurred very scarcely or not at all, so we only considered those that accounted to over 5% of the area in at least one province as potential covariates. This resulted in the four categories of evergreen needleleaf forest, deciduous needleleaf forest, mixed forests, and snow and ice being excluded.

For each time-varying variable, the annual mean and the average annual minimum and maximum levels were considered, on the basis of 4-year time series obtained for the period from 2003 to 2006. To evaluate the average annual minimum and maximum, time series were smoothed using Fourier transforms as described by Garske and colleagues [40]. The minimum and maximum of these smoothed curves determined the typical annual minimum and maximum used here. The variable that varied with time were the night- and day-time air temperatures [40], EVI, MIR [43], and rainfall [39].

Prior to fitting, all variables were scaled to unit variance in order to improve model convergence and make the fitted slope parameters comparable.

### Model Structure and Fitting

The overall model consisted of several components that were fitted jointly using standard Markov Chain Monte Carlo (MCMC) techniques [46],[47].

#### Generalised linear model for the presence/absence of yellow fever reports

A generalised linear model was fitted to the dataset describing the presence or absence of reported yellow fever over the past 25 years for each province in the countries considered endemic for yellow fever. Because of the binary nature of the data, the model used a binomial distribution with a complementary log-log link function, such that the model predictions 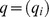 for each province 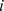 were given by
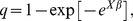
(1)where 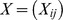 was the matrix of covariates used in the model with 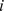 indexing provinces and 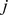 indexing the covariates, and 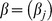 was the parameter vector to be fitted. The log-likelihood for this model component was given by
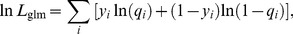
(2)where the dependent variable 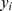 determined the presence or absence of yellow fever in province 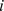 and the model predictions were considered a function of the parameter vector 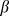, 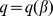. The complementary log-log link function was chosen as this could be mechanistically interpreted in terms of the surveillance quality and the actual number of infections occurring, as explained below.

As the occurrence of yellow fever certainly depends on environmental factors such as climate, land cover, but also the human population size, several environmental variables were considered as potential covariates. However, the number of such potential covariates was large, so the first step in variable selection was to fit univariate models to the dataset including each of the potential covariates in turn. Any variables that were not significantly associated with the data at the 10% confidence limit were excluded from further consideration. Some of the remaining variables were highly correlated, and inclusion of highly correlated variables in regression models can lead to instabilities in the parameter estimates. In order to avoid these problems, covariates were clustered into highly correlated groups, where the absolute pairwise correlation between any two variables within a group was above 0.75. A single variable from each group was then selected as a potential covariate in the regression modeling.

Given that the model was fitted to the presence or absence of yellow fever reports, we would also expect the quality of surveillance to have a major impact. Therefore, for all of the models considered, an indicator of the surveillance quality at the country level was also included. The parameter vector could then be expressed as 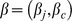, where 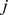 now only indexes the environmental variables included in the model (including the intercept), but not the surveillance quality, and 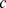 indexes the countries. From the YFSD, data on the surveillance quality for 20 countries in West and central Africa were available, defined as the per capita rate of reporting of suspect cases based on fever with jaundice as the main feature of the case definition. For these countries, the country factor was given by 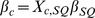, where 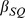 was the parameter fitted to the logarithm of the surveillance quality indicator 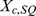, whereas for countries not covered by the YFSD, the country factor 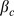 was freely fitted.

For some of the countries not covered by the YFSD, all provinces reported either presence or absence of yellow fever reports, a feature commonly found in small datasets called variable separation. Using pure maximum likelihood, this would lead to a singular estimate of the country factor [48]. To avoid any such infinite parameter estimates, for the full model framework using MCMC, Gaussian prior distributions with mean zero and standard deviation 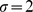 were used for all country factors. The log prior probability was given by
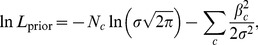
(3)where 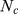 was the number of countries for which a country factor was fitted, 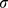 the standard deviation of the prior distributions, and 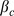 the estimate of the country factor for country 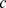.

Multivariate models were fitted using the function glm in R version 2.14.2. These models included an intercept, the log surveillance quality indicator at the country level obtained from the YFSD and a factor for each country not included in that database as well as any possible combination of up to 12 additional environmental covariates. The model fit was evaluated using the Bayesian Information Criterion (BIC) [49], and the 15 best models were further investigated in the full model framework.

#### From model predictions to transmission intensity

From the model predictions, 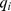, of the probability of at least one yellow fever report in province 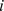 over the time period considered, the number of infections necessary to generate this probability, 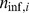 was calculated assuming a simple Poisson process for the detection of infection:
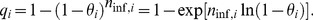
(4)Here 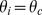 is the probability of detection of an infection, which was assumed to vary between but not within countries. Combining this with equation (1) yielded
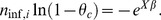
(5)By repeated taking of logarithms, this can be split into
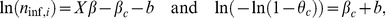
(6)where the parameter 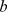 anchors the overall level of the surveillance quality relative to the transmission intensity. From the regression model alone there is not enough data to fit this parameter; additional information for estimating 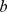 was obtained by independently fitting a force of infection to the serological survey data as described below.

The number of infections 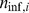 expected to accrue within province 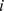 over the 25-year period covered by the incidence dataset is determined by the force of infection 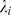 (the annual risk of infection to a susceptible person living in province 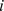), the age-specific population size, 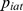, and the age-dependent vaccination coverage 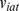, both of which varied over time 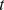:
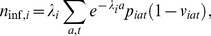
(7)which was solved numerically to estimate the force of infection 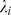 for each province 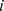 (assumed constant over time and age for simplicity).

#### Serological surveys and detection probability

Suitable serological surveys were only available for a few locations and therefore could not be used to estimate the transmission intensity across the whole endemic region. However, for the locations where serological data were available, these data were analysed to provide an estimate of the force of infection that was independent of the quality of disease surveillance [21], and could therefore be used to anchor the case detection probability to the force of infection in the regression modeling. We assumed homogeneous mixing and exposure within the survey populations and that the force of infection was independent of age but could vary between surveys. As the serological tests available cannot distinguish between natural infection and vaccination, for surveys that did not explicitly exclude vaccinated individuals, we accounted for the vaccination coverage by age at the time and location of the study. For a given force of infection 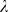, the expected seroprevalence 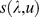 in age group 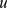 stemming from both natural infection and vaccination is given as
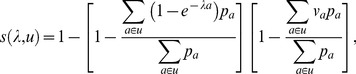
(8)where 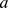 indexes the annual age groups, 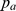 is the population age distribution, and 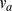 the age-dependent vaccination coverage. The log-likelihood of a given force of infection 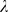, given the total number of samples tested 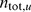 and the number of positives detected 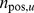 in all age groups 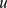 covered by the survey is then given by a binomial likelihood,
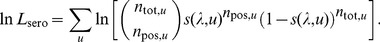
(9)For the purposes of fitting the overall model, the force of infection for each serological survey was updated in each MCMC iteration, and the log-likelihoods from all serological surveys available were summed.

The parameter 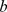 anchoring the overall level of transmission intensity was estimated using the left part of equation (6), where the parameter estimates 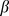 from the regression model were inserted and the expected number of infections was calculated from equation (7) using the force of infection 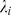 estimated from the serological surveys. This calculation yielded a different estimate of the parameter 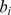 for each province covered by serological surveys, and we used the overall mean of these individual estimates as the final estimate of *b*. Inserting this estimate of 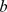 back into the right part of equation (6) then allowed the calculation of the detection probability per infection 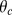 for country 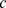 as
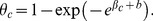
(10)The forces of infection 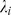 across the whole of the endemic region were then obtained by solving equation (5) for the expected number of infections 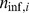, and inserting these into equation (7), which was solved numerically for each 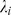.

#### Estimating the burden from transmission intensity

The annual number of infections expected in any province for any year 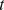 were estimated using the estimate for the force of infection 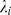 in equation (7), in conjunction with the vaccination coverage and population size at time 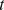. The burden for alternative vaccination coverage scenarios was obtained by refitting the model to obtain alternative estimates of the force of infection, whereas the impact of hypothetical vaccination campaigns was assessed by using the force of infection estimates from the baseline model together with vaccination coverage levels for the scenario being considered to estimate the burden under that scenario. We then assessed the impact of the vaccination scenario as the difference between baseline burden estimates and those estimated for the hypothetical scenario.

While the number of infections is the most relevant quantity for assessing the degree of transmission of yellow fever, morbidity and mortality estimates are required to assess the impact on populations and health care systems. In order to calculate the number of severe cases and deaths from the infections, we fitted beta distributions to the point estimates and 95% credibility intervals of the proportion of cases among infections and the case fatality ratio estimated by Johansson and colleagues [21] and generated samples from both distributions that we then multiplied by the number of infections estimated during each MCMC iteration. This approach allowed us to include the uncertainty of the severity spectrum in our burden estimates.

#### Model fitting

In order to obtain estimates that take into account uncertainties from all the different estimation steps, all parameters including the generalised linear regression coefficients and the force of infection estimates from the serological surveys were fitted jointly using MCMC simulations. The overall log-likelihood was the sum of the log-likelihoods from the different model components, given in equations (2), (3), and (9) as
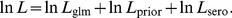
(11)To ensure a support from 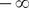 to 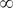 for all variables, a log transform was used for the forces of infection, which were defined on 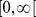, and a logit transform for any probabilities, defined on [0,1]. Proposals for the parameters on the transformed scale were then generated from a multivariate normal distribution with mean 0. To ensure optimal mixing of the chain, the standard deviations of the multivariate normal distributions were scaled proportional to the standard deviations of the posterior distribution for each parameter, as determined in some exploratory runs; these standard deviations were then scaled to yield an acceptance fraction of around 0.2 to 0.3.

For each model and value of the prior distribution standard deviation 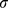 considered five chains were run for 400,000 iterations each (after burn-in), starting from different points and the results were combined. Convergence was checked visually. Prior to further analysis of the MCMC output, posterior samples were thinned by a factor of 800.

The model fit of the full model was evaluated via BIC, as this takes into account both fit quality (measured by the log likelihood) while penalizing models with a large number of parameters. In addition, we calculated receiver operator characteristic (ROC) curves comparing the regression model predictions with the yellow fever presence/absence data to which the regression models were fitted, and the area under the ROC curve (AUC), which quantifies how well the regression model predictions matched the data [50]. A lower value of the BIC indicates a better model fit, whereas a value of the AUC of 0.5 indicates that model predictions are no better than chance, and a value of 1 corresponds to a perfect fit to the data.

#### Sensitivity analyses

While the model inference framework adopted gives parameter estimates and credible intervals around these, there were however other sources of uncertainty that were more difficult to quantify, some of which were assessed in sensitivity analyses.

The impact of the choice of covariates included in the regression model was assessed by comparing the final burden estimates obtained for a number of the best fitting regression models. The model that ranked best in the initial fits of the linear regression model was used as the baseline model and is presented in the main paper, whereas results from the remaining models are shown in Text S2.

Sensitivity to the magnitude of the standard deviation of the Gaussian prior distribution on the country factors was explored (see Text S3).

The vaccination coverage dataset compiled for this study suffers from a number of uncertainties in the input datasets that are difficult to quantify, including uncertain population sizes that impact directly the vaccination coverage achieved with a given number of doses, uncertainties about the completeness and accuracy of the records of past vaccination activities, and the influence of population movements on vaccination coverage. In order to explore the potential impact of these sources of uncertainty on the burden estimates, we generated five alternative vaccination coverage scenarios: assuming only 90% vaccine efficacy, alternative lower or higher population sizes, non-random vaccine allocation, and an alternative scenario of the historic mass vaccination campaigns based on different records [51]. We used these to assess the impact of uncertainty of coverage estimates on the overall estimates of disease burden (see Text S4 for further details).

Last, we also considered two refined model structures that relaxed the assumption that the probability of case detection via routine surveillance was constant through time (see Text S5).

## Results

### Yellow Fever Occurrence

Between 1980 and 2012, 150 yellow fever outbreaks in 26 countries in Africa were reported to WHO (Figure S1). A high number of large outbreaks occurred in the late 1980s and early 1990s, particularly in Nigeria, as well as a large number of relatively smaller outbreaks in West and later central Africa since the turn of the century.

**Figure 1:**
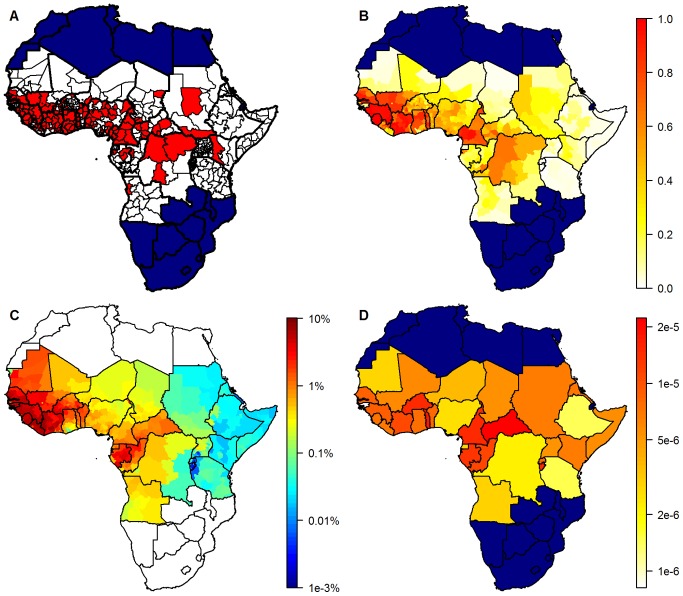
Geographical distribution of yellow fever occurrence and transmission. (A) Presence/absence of yellow fever over a 25-year period, by province. White, absence; red, presence of yellow fever reports. (B) Model predictions giving the estimated probability of at least one yellow fever report. (C) Estimates of the annual force of infection at the province level in the 32 countries considered endemic for yellow fever. (D) Estimates of the country-specific detection probability per infection. Countries not considered endemic for yellow fever are shown in navy (A, B, and D) or white (C).

The YFSD contained records of 29,237 suspected cases of yellow fever from 21 countries reported between 2005 and 2011, 302 of which were lab-confirmed, 231 classified as epidemiologically linked to a lab-confirmed case, and 416 as compatible with yellow fever based on symptoms and epidemiology, with the remaining cases considered not due to yellow fever after investigation. The locations of the lab-confirmed, linked, and compatible cases resolved to the province level are shown in Figure S2A, whereas the combined dataset of the presence or absence of yellow fever reports by province is shown in Figure 1A.

The country-specific surveillance quality (defined as the mean annual number of reported suspected cases divided by the national population) is shown in Figure S2B. While there were suspect cases reported from 21 countries, the YFSD included only five suspect cases reported from Angola, none of which were confirmed. It was therefore assumed that this country did not participate effectively in the YFSD, reducing the number of countries included to 20.

### Vaccination Coverage

The estimated vaccination coverage over time clearly shows the success of the mass vaccination campaigns in French West Africa between 1940 and 1960, and declining levels of immunity in the following decades caused by low vaccination levels, the birth of new unvaccinated cohorts, and the gradual depletion of the older protected cohorts through mortality. Between 1960 and 2000 there was limited vaccination activity across Africa resulting from disjointed reactive vaccination campaigns. Mass vaccination campaigns implemented since 2006 in the framework of the GAVI investment have achieved much higher coverage levels in West Africa (Figure S3). The impact of infant immunization on coverage at the population level will take time to develop, but if this is pursued in the future and high coverage of new birth cohorts is achieved, it will eventually lead to a high coverage even in countries with currently low population-wide coverage. In countries that currently have high population-level coverage, infant immunization will prevent a repetition of the decline in vaccination coverage observed from the 1960s onwards.

### Regression Model Fitting and Variable Selection

All models included log[surveillance quality] and country factors for those countries for which surveillance quality data were not available (due to non-participation in YFSD). In addition a total of 34 potential covariates were evaluated, nine of which were not significantly associated with the data at the threshold of *p* = 0.1 (see Table S2). The remaining 25 variables were clustered into 18 groups (see Figure S4 for the correlations between variables and Figure S5 for maps of the 18 covariates considered in the multivariate regression models), leading to a total of 249,527 models fitted with standard regression software. The 15 best models further investigated in the full model framework included three to five additional covariates (Table 1). These models were investigated further by MCMC, fitting simultaneously the regression parameters and the force of infection from the serological surveys. For identification, these models were indexed with their BIC rank from the initial model fit. Time series and autocorrelation plots of the model parameters for the baseline model (model 1) are shown in Figures S6 and S7, respectively.

**Table 1:** Covariates included in the 15 models investigated, ordered by rank of the Bayesian Information Criterion from the initial fits of the regression model only.

Model Index	Number of Variables	Log(population)^a^	Longitude	Latitude	Mean EVI	Mean MIR	Deciduous Broadleaf Forest	Open Shrubland	Cropland/Natural Vegetation Mosaic	Barren Or Sparsely Vegetated
1	4	1	1	0	1	0	0	0	1	0
2	3	1	1	0	1	0	0	0	0	0
3	3	1	1	0	0	1	0	0	0	0
4	5	1	1	1	1	0	0	0	1	0
5	3	1	1	0	0	0	0	1	0	0
6	4	1	1	1	0	1	0	0	0	0
7	4	1	1	0	0	1	0	0	1	0
8	4	1	0	1	1	1	0	0	0	0
9	3	1	0	1	1	0	0	0	0	0
10	5	1	1	1	0	1	0	0	0	1
11	4	1	0	1	1	0	0	0	1	0
12	3	1	0	1	0	1	0	0	0	0
13	5	1	1	0	1	0	1	0	1	0
14	4	1	0	1	0	1	0	0	0	1
15	5	1	1	0	1	0	0	1	1	0

a 1, variable included; 0 = variable not included.

One would expect the number of cases to be proportional to the population size, leading to a dependence of the model predictions (probability of detecting yellow fever) on the log[population size], and this covariate was indeed included in all of the 15 best fitting models with a regression parameter value around 1, indicating linear dependence of the number of cases on population size. Most models included longitude, mimicking the strong gradient in risk that is observed in yellow fever epidemiology. Latitude, mean EVI, mean MIR, and the land cover category indicating a mosaic of cropland and natural vegetation were included in about half of the models, with typically each model including either mean EVI or mean MIR. The land cover categories of deciduous broadleaf forest, open shrubland, and barren areas were only included in few models, and no further of the 18 potential covariates considered were included in the 15 best fitting models.

**Figure 2:**
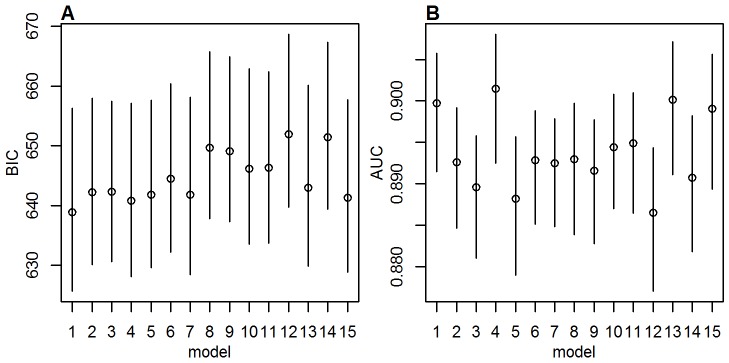
Goodness of fit measures. (A) BIC and (B) AUC values for the 15 models investigated with MCMC, with a prior standard deviation on the country factors of 2. Circles show posterior means, lines the 95% posterior range.

The differences in goodness of fit between the models were small compared with the uncertainty inherent in the BIC and AUC estimates (Figure 2), although the BIC indicated a slightly better fit for the models with a smaller index in Table 1 compared with those with a larger index, mirroring the BIC rank in the pure regression models. AUC values were high, averaging just below 0.9, showing a good match between data and regression model predictions. The AUC indicated a better match between regression model predictions and data for models 1, 4, 13, and 15 than the other models, but again, the differences were small.

As the model fit and burden estimates obtained with the different models were similar, for the remainder of this manuscript only results from model 1 are presented, with a standard deviation of the prior distributions of 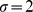. For results from the other models and extensive sensitivity analyses see Texts S2, S3, S4, S5.

### Outputs from the Baseline Model

The high values for the AUC seen for the model predictions testified to a good model fit, so it is unsurprising that the spatial distribution of the model predictions matched the dataset of presence or absence of yellow fever reports very well (Figure 1A and 1B). The model successfully captures the gradient of transmission intensity from west to east as well as the focus of transmission being in sub-Sahel and tropical latitudes, which is reflected in both the model predictions (Figure 1B) and the force of infection estimates (Figure 1C).

There was substantial uncertainty in the force of infection estimates, with the highest values of the coefficient of variation being in areas with the lowest force of infection estimates: Rwanda, Burundi, and western parts of Tanzania (Figure S8). Due to the very low force of infection estimates in these areas, this uncertainty has little impact on the burden estimates.

The estimated country-specific detection probability per infection varied over nearly two orders of magnitude between countries. Countries with a higher estimated force of infection also had higher estimates of the case detection probability, with the highest values found in the Central African Republic and Togo and the lowest in Guinea-Bissau, Ethiopia, and Tanzania (Figure 1D), Notably the detection probability was estimated to be very low in Nigeria, which has a substantial impact on the burden estimates due to its large population.

The annual number of yellow fever infections, severe clinical cases, and deaths expected from the estimated force of infection were estimated for selected years (Table 2). Between 1995 and 2005, the overall vaccination coverage remained roughly similar across the continent. The moderate increase in estimated burden between these years therefore reflects overall population growth. However, the large preventive mass vaccination campaigns performed between 2006 and 2012 increased the vaccination coverage in the participating countries, substantially outweighing population growth effects and resulting in a 2013 burden estimate of 180,000 (95% CI 51,000–380,000) severe cases presenting with fever and jaundice or haemorrhage including 78,000 (95% CI 19,000–180,000) deaths. We estimate that the recent preventive mass vaccination campaigns between 2006 and 2012 reduced the annual burden evaluated for 2013 by 27% (95% CI 22%–31%), which equates to an overall reduction of 57% (95% CI 54%–59%) in the 12 targeted countries. In these campaigns, the number of targeted provinces and districts and therefore the impact achieved varied by country, with the highest reductions achieved in Benin, Togo, and Cote d'Ivoire, where an estimated 82%, 77%, and 73%, respectively, of the burden was prevented in 2013. The reduction at the national level of participating countries reflects both vaccinated and non-vaccinated regions within each country.

**Table 2:** Estimated burden in terms of the number of infections, severe cases, and deaths (95% CIs) due to yellow fever in Africa for three selected years, and the estimated burden averted for 2013 (95% CIs) due to preventive mass vaccination campaigns in Africa from 2006 to 2012.

Year	Number of Infections	Number of Severe Cases	Number of Deaths
1995	1,500,000 (1,100,000–2,200,000)	220,000 (63,000–470,000)	95,000 (24,000–220,000)
2005	1,800,000 (1,200,000–2,500,000)	250,000 (73,000–530,000)	110,000 (27,000–250,000)
2013	1,300,000 (850,000–1,800,000)	180,000 (51,000–380,000)	78,000 (19,000–180,000)
Averted in 2013 by preventive campaigns	450,000 (340,000–560,000)	63,000 (19,000–130,000)	28,000 (7,200–62,000)

**Figure 3:**
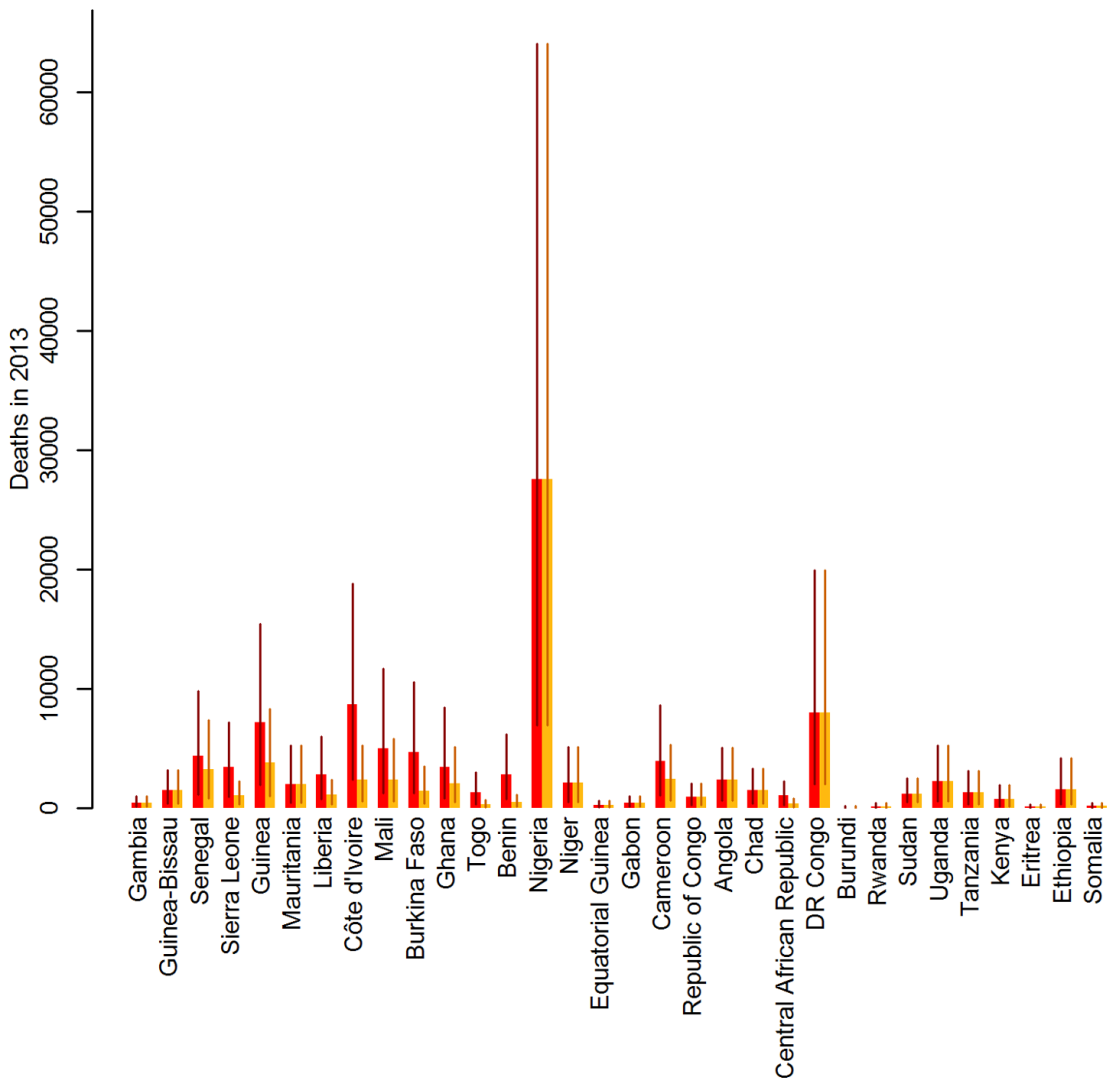
Impact of preventive mass vaccination campaigns between 2006 and 2012 on the estimated number of deaths due to yellow fever in 2013 by country. Red bars show the number of deaths estimated assuming implementation of no mass vaccination campaigns between 2006 and 2012, orange bars show the number of deaths estimated for the actual vaccination. Lines show the 95% credibility intervals of the estimated number of deaths. Countries are ordered west to east.

Disease burden was estimated to be distributed very unevenly between countries, with by far the largest burden estimated for Nigeria, owing to the moderately high force of infection, low vaccination coverage, and a large population size (Figure 3). The country contributing the next largest number of cases and deaths was the Democratic Republic of the Congo, followed by countries in West Africa with a high force of infection, some of which have recently benefited from the GAVI-funded mass vaccination campaigns (Table 3).

**Table 3:** Number of yellow fever vaccine doses procured for use in preventive vaccination campaigns from 2006 to 2012, percentage of the national population targeted and burden reduction achieved in 2013, by country.

Country^a^	Vaccine Doses (Millions)	Percent Population Targeted	Percent Burden Reduction (95% CI)
Senegal	3.1	28	26 (22–30)
Sierra Leone	4	71	68 (67–71)
Guinea	6	61	47 (45–50)
Liberia	2.9	78	60 (58–62)
Côte d'Ivoire	18.8	98	73 (69–77)
Mali	5.9	42	53 (48–57)
Burkina Faso	7.6	50	69 (66–71)
Ghana	7.6	32	38 (36–40)
Togo	3.6	65	77 (74–79)
Benin	8.2	98	82 (81–83)
Cameroon	7.5	40	38 (37–40)
Central African Republic	2.6	61	64 (64–65)

a Countries are ordered west to east.

**Figure 4:**
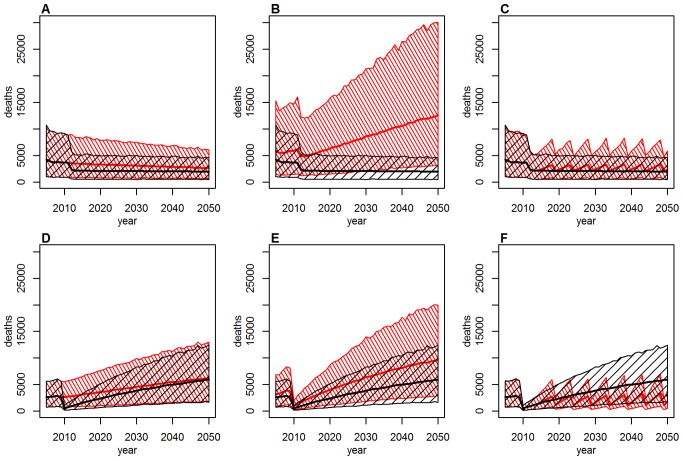
Deaths over time under various vaccination coverage scenarios for Ghana (top: A–C) and Liberia (bottom: D–F). Thick lines show the point estimate, hashed areas the 95% credibility intervals. Baseline scenario (black) in (A–F) includes past mass vaccination and infant immunization, plus continuing infant immunization at 2011 coverage levels. Alternative scenario (red): (A and D): as baseline, but excluding the mass vaccination campaigns; (B and E): as baseline, but assuming no infant immunization at any time; (C and F): as baseline, but including mass vaccination campaigns targeting children under 5 every 5 years at a coverage of 90% instead of future infant immunization.

Mass vaccination campaigns can be extremely effective at reducing the burden in populations with low immunity, with the effect being immediate and long lasting (Figure 4). The impact wanes over the course of decades only as new birth cohorts join the populations (Figure 4A and 4D). In this context, routine infant immunization as performed in the EPI in many African countries since the 1980s (Table S1) serves an important purpose by ensuring good vaccination coverage in new birth cohorts and thus preventing any long term decrease in population immunity. As the sole tool to increase population immunity infant immunization is less effective, as it takes decades for such a program to substantially increase the immunity of the whole population. Figure 4B and 4E shows the burden in Ghana and Liberia assuming no infant immunization ever in these two countries. The results illustrate how a high infant immunization coverage is crucial to sustaining low levels of burden (as in Ghana with 91% coverage), whereas low coverage levels (as in Liberia with 39% coverage) will reduce the burden a little but are too low to sustain a low level of burden in the future. A combination of mass vaccination campaigns and infant immunization at good coverage level is therefore likely to reduce the burden quickly and sustain it at low levels.

Some countries achieve high coverage in their routine infant immunization but the coverage in other countries is low. Conversely, the mass vaccination campaigns achieved high coverage levels in most countries targeted. If it is difficult to reach a substantial proportion of infants with routine immunization, one could instead consider repeated mass vaccination campaigns. Figure 4C and 4F shows the effect of repeating mass vaccination campaigns targeting children under 5 every 5 years is similar to what is achieved using routine infant immunization reaching a high proportion of infants. Such age-targeted campaigns would cost less than repeated mass vaccination campaigns targeting all age groups while being similarly effective.

**Figure 5:**
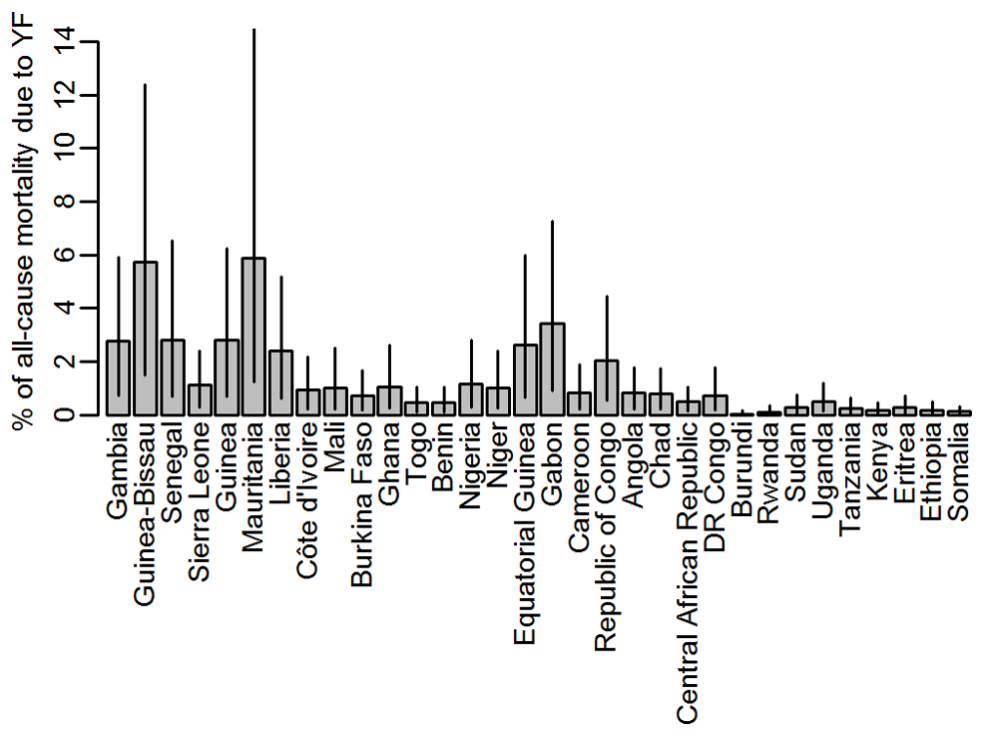
Percentage of the all-cause mortality attributable to yellow fever by country. Grey bars indicate the point estimates, black lines the range spanned by the 95% CIs of the burden estimates. Countries are ordered west to east.

We compared our estimates of mortality due to yellow fever to all-cause crude mortality estimates obtained from the UN WPP [35] for all endemic countries. For the period from 2005 to 2010, the estimates varied between eight and 18 deaths per year per 1,000 population, equating to 9.4 million deaths annually from any cause in the endemic region (calculated using 2010 population estimates). Our estimate of 78,000 deaths from yellow fever for 2013 therefore corresponds to 0.8% of all-cause mortality, but the proportion of the all-cause mortality that would be attributed to yellow fever based on our burden estimates varied substantially between countries (Figure 5), ranging from close to zero in many east African countries to values typically between 1% and 3% in West Africa, with the highest values just under 6% in Mauritania and Guinea-Bissau.

## Discussion

In this study, we estimated the burden of yellow fever in terms of the number of infections, severe cases, and deaths across Africa by fitting generalised linear regression models to datasets of yellow fever reports between 1987 and 2011. We evaluated the impact of recent large-scale preventive mass vaccination campaigns undertaken between 2006 and 2012 under the Yellow Fever Initiative by estimating the burden expected had these vaccination campaigns not taken place.

We estimate that currently there are between 51,000–380,000 severe cases of yellow fever annually in Africa, resulting in an estimated 19,000–180,000 deaths. These figures are to be compared with previous global estimates of 200,000 cases and 30,000 deaths annually for the early 1990s, around 90% of which occur in Africa [5],[6],[52]. It is encouraging that both sets of estimates are broadly similar, particularly since the new estimates take into account all existing data on yellow fever that are currently available. The analysis provided here also gives a better understanding of the spatial and temporal distribution of yellow fever across Africa. The model framework developed takes into account a variety of different data sources, including information on population vaccination coverage over time, which can be used to evaluate the impact of past and potential future vaccination campaigns.

The average annual number of yellow fever cases officially reported to WHO by countries in the endemic zone [53] was 1,165 for the period from 1987 to 2011 considered in this analysis, and 656 for the period between 2005 and 2011 covered by the YFSD (note that this is a different dataset than the YFSD, containing only aggregate numbers). This was in contrast to the estimated annual burden of around 180,000 severe cases (which were defined as presenting with fever and jaundice or haemorrhage), meaning that for each officially reported case there might actually be as many as 50 to 500 severe cases. This is consistent with the 10–1,000-fold under-ascertainment of yellow fever morbidity and mortality recognized in past work [3],[4]. Such levels of under-ascertainment highlight the difficulties inherent in yellow fever surveillance, which relies on clinical case definitions. Syndromic surveillance is challenging due to the variety of clinical manifestations seen in severe disease that do not include jaundice and therefore might be mistaken for other infections (notably malaria) [26]. In addition, not all jaundice is caused by yellow fever, with other causes including malaria, liver pathogens, and other conditions.

The detection probabilities fitted in our model are of the order of 10^−5^, but these describe the probability that an infection would be reported into either the YFSD or as an outbreak. In the YFSD, there were on average around 135 cases reported annually. Comparing this to our burden estimates of around 1.5 million infections annually in the time period covered by the YFSD, this would lead to an empirical detection probability of the order of 10^−4^ across Africa, an order of magnitude larger than the values fitted in our model. However, the detection probabilities fitted in our model represent an average over 25 years, and detection was considerably poorer prior to the introduction of the YFSD.

The proportion of the all-cause mortality that would be attributed to yellow fever based on our burden estimates varied between countries with plausible estimates of less than 3% for most countries, with the exception of Mauritania and Guinea Bissau where nearly 6% of the all-cause mortality would be attributed to yellow fever on the basis of our estimates. The estimates for these two countries may appear unrealistically large, but it should be kept in mind that the uncertainty in the force of infection and consequently in the burden estimate is relatively high in Mauritania (Figures 3 and S7), whereas for Guinea-Bissau, the estimated detection probability is the lowest estimated for any country (Figure 1D) due to its low rate of reporting suspected cases to the YFSD. If the overall surveillance quality in this country was not well represented by the participation in the YFSD, the burden estimate here would be over-inflated.

The datasets of yellow fever incidence used to fit the models rely on surveillance recognizing yellow fever cases. Typically the case definition is based on fever with jaundice and/or haemorrhaging symptoms, but of course the sensitivity and specificity of this case definition might vary between settings. In our analysis, we have allowed for the sensitivity to vary between countries by estimating the country-specific surveillance quality. The specificity of the case definition in our datasets should be high across the board, as only laboratory confirmed cases or cases closely linked epidemiologically were included in our analysis. There might however be substantial differences in the severity spectrum of yellow fever between settings, depending on factors such as previous exposure to other flaviviruses, the general immune status of the populations, or the access to health care facilities, although there is no treatment for yellow fever apart from general life support. While we were not able to include any of these effects, we used estimates of the severity with measures of uncertainty by Johansson and colleagues based on the limited available data [21], capturing the variability seen across different settings. Our model estimates first the number of infections and infers the disease burden in terms of the number of severe cases and deaths from this, so the uncertainty of our burden estimates is inflated by the uncertainty of the severity spectrum. Nevertheless we have chosen to report mainly the number of deaths as cases and deaths are more relevant in terms of disease burden and health care needs than the number of infections, the majority of which are likely to be very mild or asymptomatic.

The credible intervals around the burden estimates presented here also reflect the fact that a range of values for the force of infection estimates yield a similarly good model fit. However, while the credible intervals represent the uncertainty in model parameter estimates, there are further potential sources of uncertainty that are not captured by credible intervals. Firstly, the choice of covariates included in the model could have an effect. However, the 15 models investigated here showed a similarly good fit to the dataset, and the burden estimates from all models were very similar (see Text S2).

Second, in order to prevent the country factors (which determine the detection probabilities in countries not participating in the YFSD) from taking infinite values, we assumed a Gaussian prior distribution for these within the Bayesian framework used for model fitting. The standard deviation of this prior distribution was chosen relatively arbitrarily; however, in the sensitivity analyses we have shown that the burden estimates again are fairly independent of the particular value chosen (see Text S3).

The dataset of vaccination coverage compiled from various sources reporting on vaccination activities in the last century contains a number of potential sources of uncertainty that are very difficult to quantify. Uncertainty in historical population sizes by age generates uncertainty in vaccination coverage estimates if those estimates are generated from records of the number of vaccine doses used. There are also concerns about the completeness and accuracy of the reports on vaccination activity. Furthermore, the effect of population movements on vaccination coverage could not be taken into account owing to lack of data. Our simplifying assumptions of a 100% vaccine efficacy and lifelong immunity conferred by the vaccine can also be questioned. To evaluate the impact of these uncertainties we undertook sensitivity analyses that carried vaccine effectiveness and the coverage achieved in historical campaigns, but found the effects on burden estimates to be slight (see Text S4). While we omitted reactive vaccination campaigns before 1970 in the generation of all vaccination coverage scenarios as these data were not routinely reported prior to this time, this is likely to have little impact on vaccination coverage levels due to the low yellow fever activity and resulting low number and extent of vaccination campaigns in this period.

Uncertainty in demographic data across Africa has a very direct impact on the burden estimates, as such estimates are directly proportional to the population size. This uncertainty is not captured in the confidence intervals given in this paper, as it was not possible to quantify the level of uncertainty.

There are substantial uncertainties regarding the spatial distribution of yellow fever occurrence, which were taken into account in our model by allowing infection risk and detection probabilities to vary between countries. However, the baseline model presented above did not allow for detection probabilities to vary over time, while activities such as the introduction of the YFSD in 2005 were clearly intended to improve surveillance. We therefore investigated two alternative model structures that both allow for a change in the detection probabilities at the time of introduction of the YFSD, both of which estimated an increased probability of case detection in the countries participating in the YFSD following its introduction. The overall burden estimates from these models were very similar to those obtained from the baseline model though there were subtle differences in the spatial distribution of the transmission intensity, with one of the alternative models showing a slightly less pronounced gradient in transmission strength from west to east (see Text S5).

Similarly, while we allowed the force of infection for yellow fever to vary in space, we assumed it was constant throughout the 25-year observation period, as well as homogeneous by gender and age. While clearly there will be differences in exposure between age groups and genders, particularly in areas where non-human primates play an important role in transmission, the relatively crude nature of the yellow fever occurrence data did not support a model that would be able to estimate these differences. Our estimates are therefore representative of the overall population but do not reflect the age- and sex-specific exposure likely to be found in many places. The assumption of constant force of infection throughout time means we have not taken into account changes in transmission due to factors such as changed land use or climate change, which might influence the transmission intensity. Clearly yellow fever activity is not constant, but epidemic amplifications and reductions of transmission intensity happen over the timescale of decades. Epidemics are driven, at least in part, by the rapid removal and slow replenishment of susceptible hosts in both humans and wildlife, as illustrated by the widespread epidemics in much of western Africa, and particularly Nigeria, in the 1990s, and a reduction in epidemic activity since then. Furthermore, a serological survey in Central African Republic testing samples collected in 2006 and in 2009 found evidence of an increase in yellow fever exposure over this period [29], mirroring the increasing number of cases reported from that region in recent years. Therefore our results should be seen as representative of the past 25 years, averaging over the large fluctuations that occur in reality, although the burden estimates for specific years do reflect the population size, age structure, and vaccination coverage pertaining to the time.

Burden estimates were strongly determined by the force of infection estimated from serological surveys [29]–[34]. However, the only surveys available were conducted in central Africa and Nigeria, with these results extrapolated to the remainder of the endemic zone in West and East Africa using the spatial distribution of transmission intensity estimated from the regression model. While all model structures reproduced the gradient in transmission intensity from west to east that is seen in yellow fever epidemiology, this gradient was more pronounced in the baseline model presented in the main paper than in the alternative model that was fitted to an annual dataset of yellow fever reports (see Text S5). In the absence of further reliable serological data outside central Africa it is presently not possible to distinguish which model better reflects reality. There are several serological surveys under way or close to completed in east African countries including Sudan, Rwanda, Uganda, Kenya, South Sudan, and Ethiopia. These data, once available, will substantially reduce model uncertainty, allowing us to discriminate between different model assumptions and resulting in more reliable estimates.

Cohort studies collecting data on case incidence and the severity spectrum of disease could also reduce the level of uncertainty. The relatively low incidence of yellow fever implies the need for large cohorts, which would be prohibitively expensive if performed for yellow fever alone. However, including yellow fever diagnostics into ongoing cohort studies (e.g., focused on HIV or malaria) might be a cost-effective way to improve basic understanding of yellow fever epidemiology. A further advantage of studies focusing on multiple diseases would be to understand interactions between infections (most notably cross-immunity between flaviviruses).

Our analysis does not take into account the epidemic character of yellow fever transmission, but rather assumes cases are distributed evenly over time according to a force of infection that is independent of the incidence of cases in the population. Consequently, the impact of vaccination campaigns will be underestimated, as lower transmission in a population due to vaccination also provides indirect protection to unvaccinated individuals (herd immunity). While the impact of herd immunity can be easily quantified in situations where there is only one type of host, this is currently impossible with yellow fever as it is unknown what proportion of cases arise through inter-human transmission via mosquito vectors, and what proportion through the sylvatic cycle. While this question cannot be answered with the methodology employed in the present study, it is an important topic for future work.

Keeping this limitation in mind, we conservatively estimate that the recent mass vaccination campaigns have reduced the yellow fever burden in the 12 participating countries for 2013 by 57% (95% CI 54%–59%) relative to a counterfactual scenario in which these campaigns were not conducted, by vaccinating 78 million people, who make up around 55% of the population of these countries. Across Africa, this amounts to a reduction of the total burden of yellow fever by 27% (95% CI 22%–31%), by vaccinating around 10% of the population in the endemic zone.

Partly as a result of the estimates presented here, in late 2013 the GAVI Alliance Board decided to make available support for additional yellow fever vaccination campaigns, targeting 144 million people across the endemic region in Africa [54],[55]. Furthermore, the GAVI Alliance is now using our estimates for evaluating the past and future impact of their yellow fever vaccination activities.

The impact of both past and future mass vaccination campaigns will prevent a substantial proportion of yellow fever disease burden for years to come, with a gradual decrease in impact over the next decades as new birth cohorts that have not benefitted from these campaigns enter the population. This effect of slowly declining vaccination coverage following the abandonment of mass vaccination campaigns was seen since the 1960s, and was the cause of the gradual resurgence of yellow fever over the following decades. However, the achievements of the current mass vaccination campaigns could be sustained if a high level of immunization is achieved through a strong EPI program and preventive vaccination of populations that remain at-risk, such as migrants or populations from as yet unvaccinated districts. While the coverage achieved in the routine infant immunization is variable between countries, the coverage achieved in recent mass vaccination campaigns has generally been high. An alternative for countries struggling to reach high EPI coverage levels might therefore be to repeat mass vaccination campaigns targeted at children every few years, although the organizational and financial costs would probably be substantially higher than the existing EPI.

Yellow fever is a disease that is difficult to diagnose and confirm, whose symptoms can be mild and mistaken for other infections, and that occurs in some of the most resource-poor settings globally. Consequently surveillance data reflect patterns of endemicity and emergence of infection in new zones and provide sentinel data on imminent or ongoing outbreaks, but do not reflect the actual disease burden. The most recent estimates of the disease burden stemmed from the early 1990s and therefore an update taking into account the changes in demography, ecology, and vaccination coverage, such as the estimates provided in the present study, was long overdue. The framework for burden estimation developed here is also a useful tool for the evaluation and planning of effective vaccination campaigns. As such, it is being used by the partners of the Yellow Fever Initiative for planning their yellow fever vaccination strategy for the next decade.

## Supporting Information

Figure S1 **Map of the outbreaks recorded in Africa between 1980 and 2012.** Outbreak size indicated by the symbol size, outbreak year coded by the colour.
(PNG)

Figure S2 **(A) map of the number lab-confirmed, epi-linked, and compatible yellow fever cases reported in the YFSD by province.** (B) Annual reporting rate of suspected cases per 100,000 population by country.
(PNG)

Figure S3 **Estimated vaccination coverage at the first administrative level in the countries endemic for yellow fever on the African continent throughout the decades.** Non-endemic countries are shown in grey. The estimate for 2015 is a projection that assumes infant immunization continues at the same levels as in 2011, and no other vaccination campaigns are implemented.
(PNG)

Figure S4 **Absolute values of the pairwise correlations between the 25 potential covariates significant at the** ***p*** **= 0.1 level from 0 (red) to 1 (white).** Clusters are highlighted by a lack of separating lines, and variables not considered for the multivariate models printed in grey.
(PNG)

Figure S5 **Maps of the 18 variables considered in the multivariate modeling as potential covariates.** Colour scale from navy (low) to red (high). A, longitude; B, latitude; C, altitude; D LC, deciduous broadleaf forest; E LC, closed shrubland; F LC, open shrubland; G LC, woody savannas; H LC, urban and built-up; I LC, cropland/natural vegetation mosaic; J LC, barren or sparsely vegetated; K, mean day temperature; L, min day temperature; M, min night temperature; N, max night temperature; O, max EVI; P, min MIR; Q, min rainfall; R, max rainfall.
(PNG)

Figure S6 **MCMC posterior trace plots of model parameter estimates for the baseline model, thinned by a factor 800.**
(PNG)

Figure S7 **Auto-correlation in posterior estimates of the model parameters for the baseline model.** Posterior MCMC samples were thinned by a factor 800.
(PNG)

Figure S8 **Coefficient of variation of the force of infection estimates.** Countries not considered endemic for yellow fever are shown in white.
(PNG)

Table S1 **Coverage and year of introduction of the yellow fever vaccine into the routine Enhanced Programme of Immunization by country.**
(PDF)

Table S2 **Covariates considered in the regression modeling, significance level in univariate models and cluster association.**
(PDF)

Text S1 **Demographic data analysis.**
(PDF)

Text S2 **Sensitivity analysis: impact of the covariates included.**
(PDF)

Text S3 **Sensitivity analysis: impact of the standard deviation of the prior distribution on the country factors.**
(PDF)

Text S4 **Sensitivity analysis: impact of alternative vaccination coverage scenarios.**
(PDF)

Text S5 **Sensitivity Analysis: alternative model structures.**
(PDF)

## Abbreviations

AUC: area under the curve
BIC: Bayesian Information Criterion
EPI: Enhanced Programme for Immunization
EVI: enhanced vegetation index
GAVI: Global Alliance for Vaccines and Immunization
MCMC: Markov Chain Monte Carlo
MIR: middle infrared reflectance
YFSD: yellow fever surveillance database
