# Supplementary figures and images for: Yellow Fever in Africa: Estimating the Burden of Disease and Impact of Mass Vaccination from Outbreak and Serological Data
Source: PLoS Med. 2014 May 6;11(5):e1001638. doi: 10.1371/journal.pmed.1001638 (PMC4011853; doi:10.1371/journal.pmed.1001638)

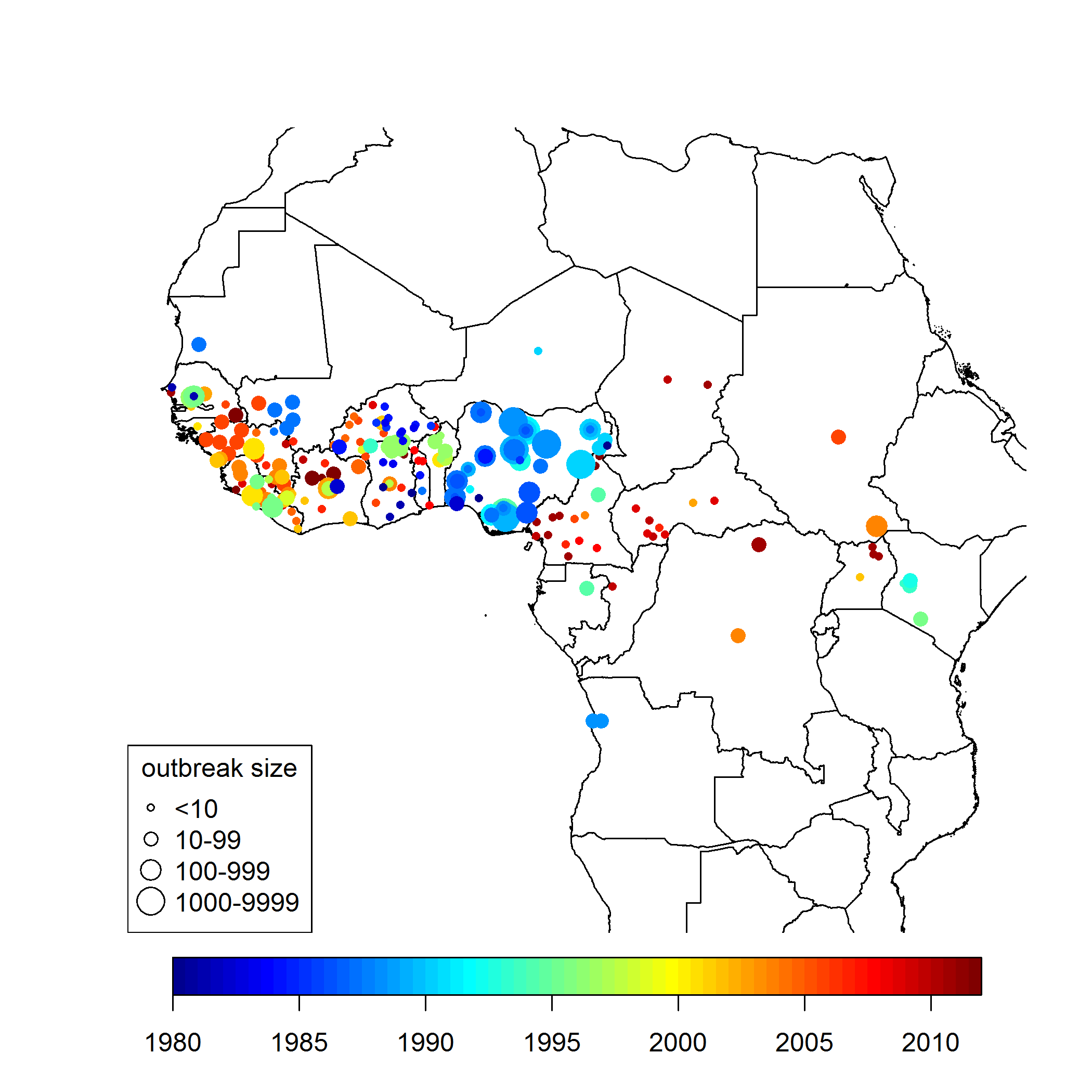

Supplement: Figure S1 — Map of the outbreaks recorded in Africa between 1980 and 2012. Outbreak size indicated by the symbol size, outbreak year coded by the colour. (PNG) [file pmed.1001638.s001.png]

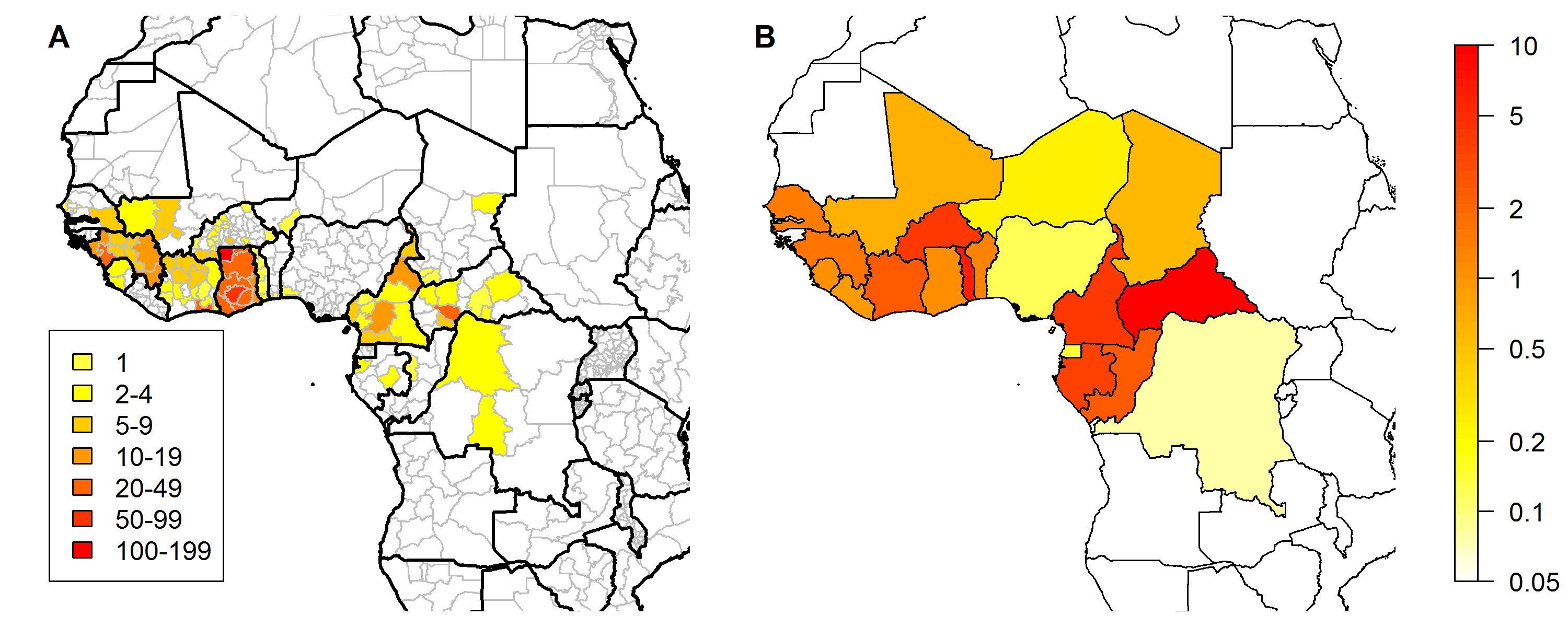

Supplement: Figure S2 — (A) map of the number lab-confirmed, epi-linked, and compatible yellow fever cases reported in the YFSD by province. (B) Annual reporting rate of suspected cases per 100,000 population by country. (PNG) [file pmed.1001638.s002.png]

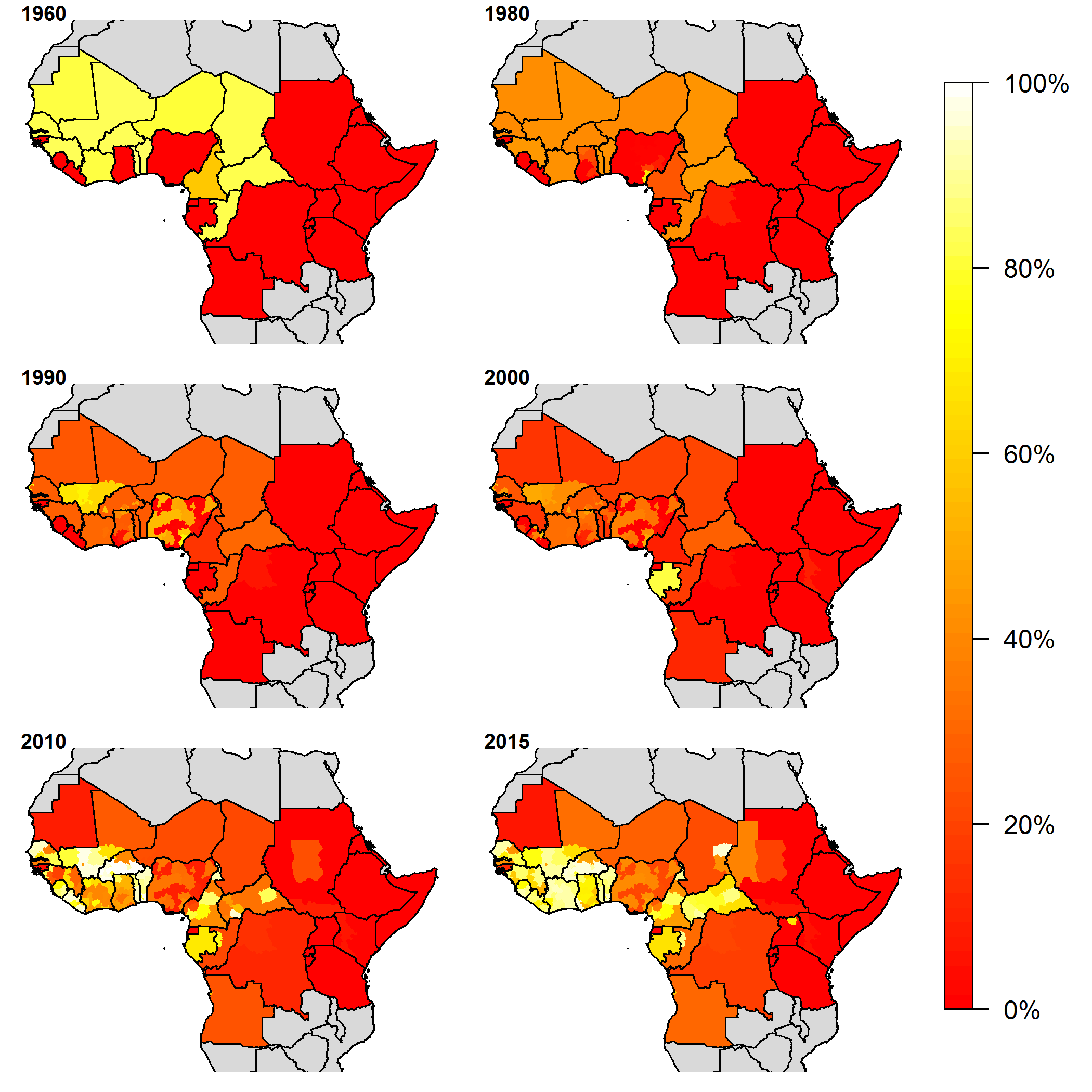

Supplement: Figure S3 — Estimated vaccination coverage at the first administrative level in the countries endemic for yellow fever on the African continent throughout the decades. Non-endemic countries are shown in grey. The estimate for 2015 is a projection that assumes infant immunization continues at the same levels as in 2011, and no other vaccination campaigns are implemented. (PNG) [file pmed.1001638.s003.png]

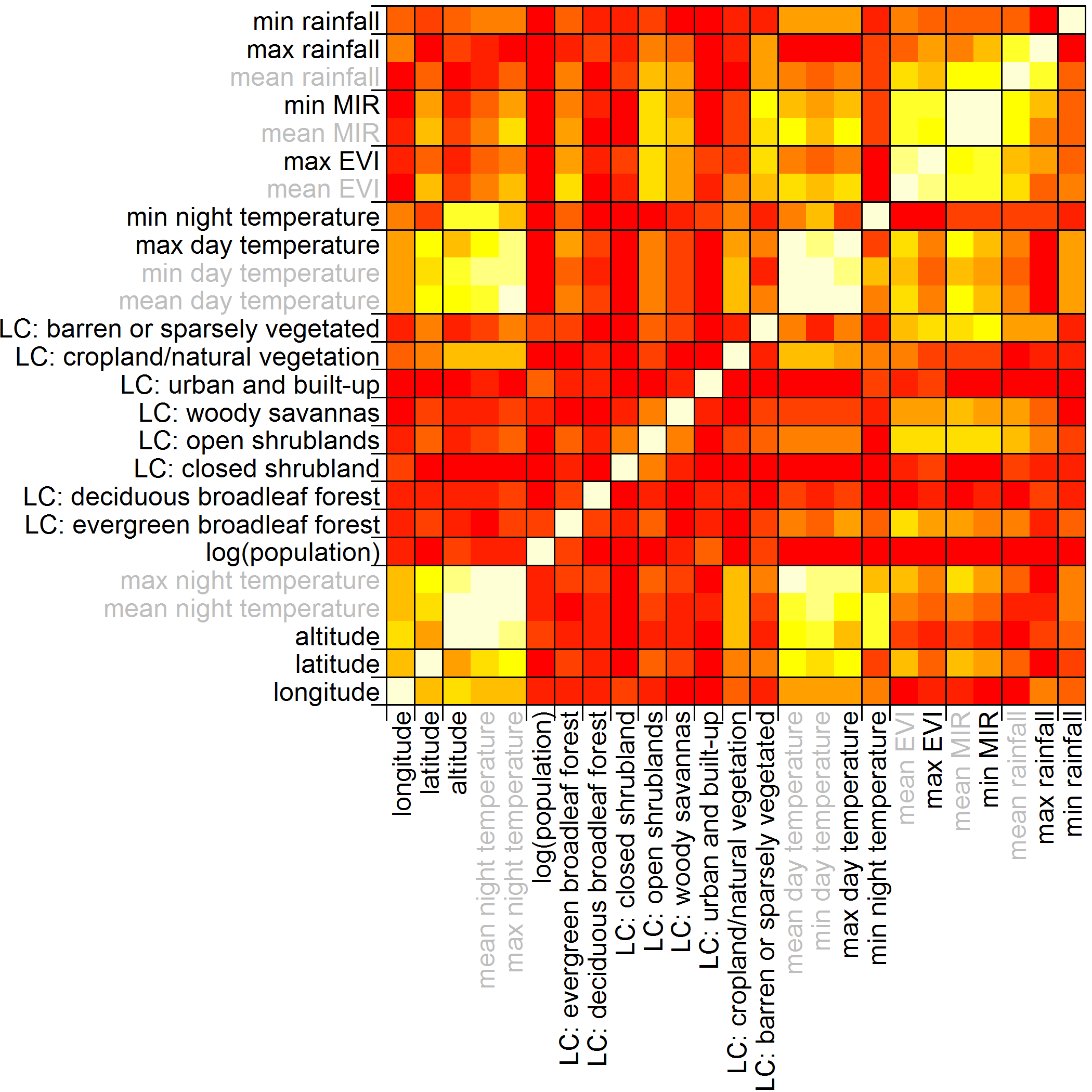

Supplement: Figure S4 — Absolute values of the pairwise correlations between the 25 potential covariates significant at the p = 0.1 level from 0 (red) to 1 (white). Clusters are highlighted by a lack of separating lines, and variables not considered for the multivariate models printed in grey. (PNG) [file pmed.1001638.s004.png]

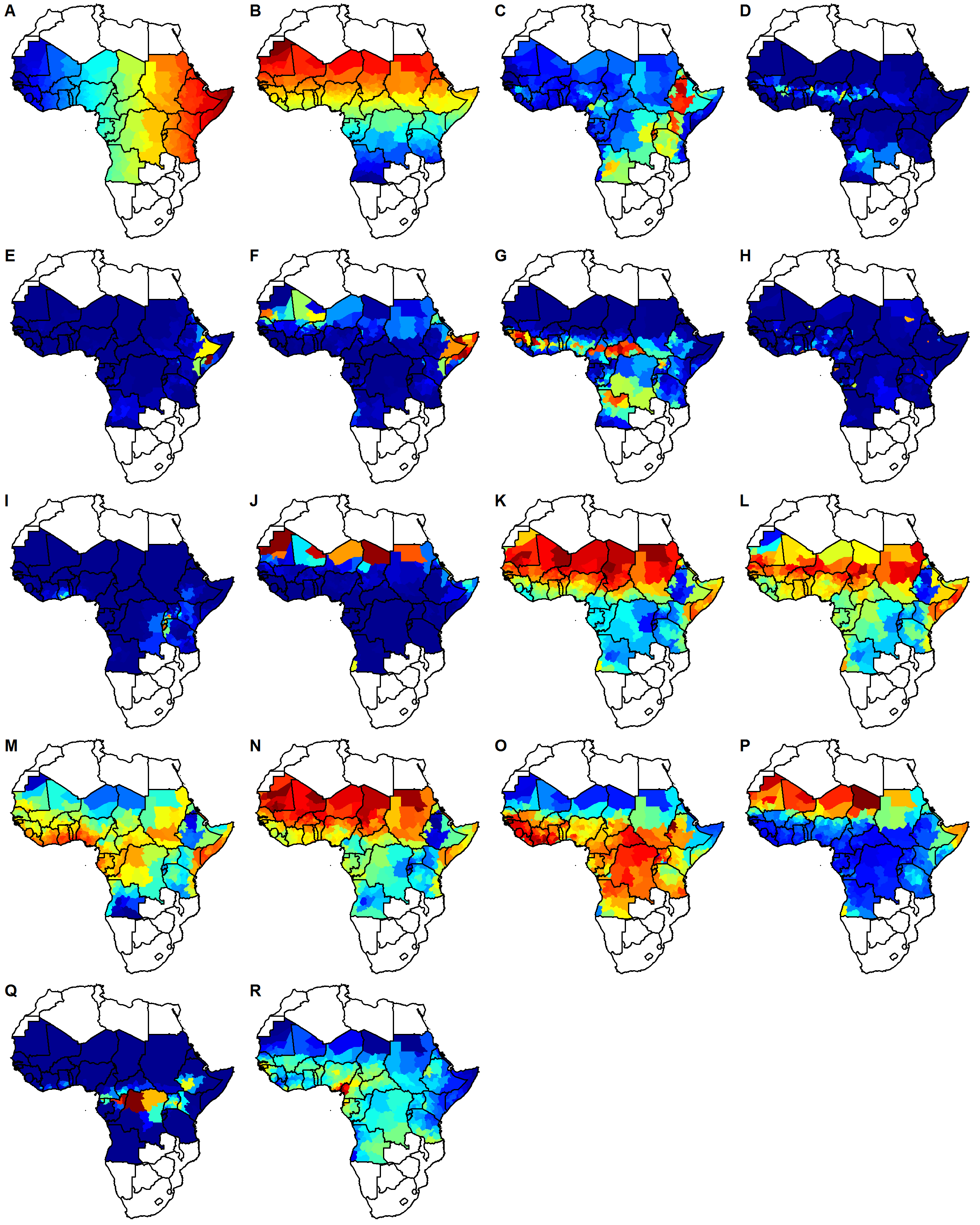

Supplement: Figure S5 — Maps of the 18 variables considered in the multivariate modeling as potential covariates. Colour scale from navy (low) to red (high). A, longitude; B, latitude; C, altitude; D LC, deciduous broadleaf forest; E LC, closed shrubland; F LC, open shrubland; G LC, woody savannas; H LC, urban and built-up; I LC, cropland/natural vegetation mosaic; J LC, barren or sparsely vegetated; K, mean day temperature; L, min day temperature; M, min night temperature; N, max night temperature; O, max EVI; P, min MIR; Q, min rainfall; R, max rainfall. (PNG) [file pmed.1001638.s005.png]

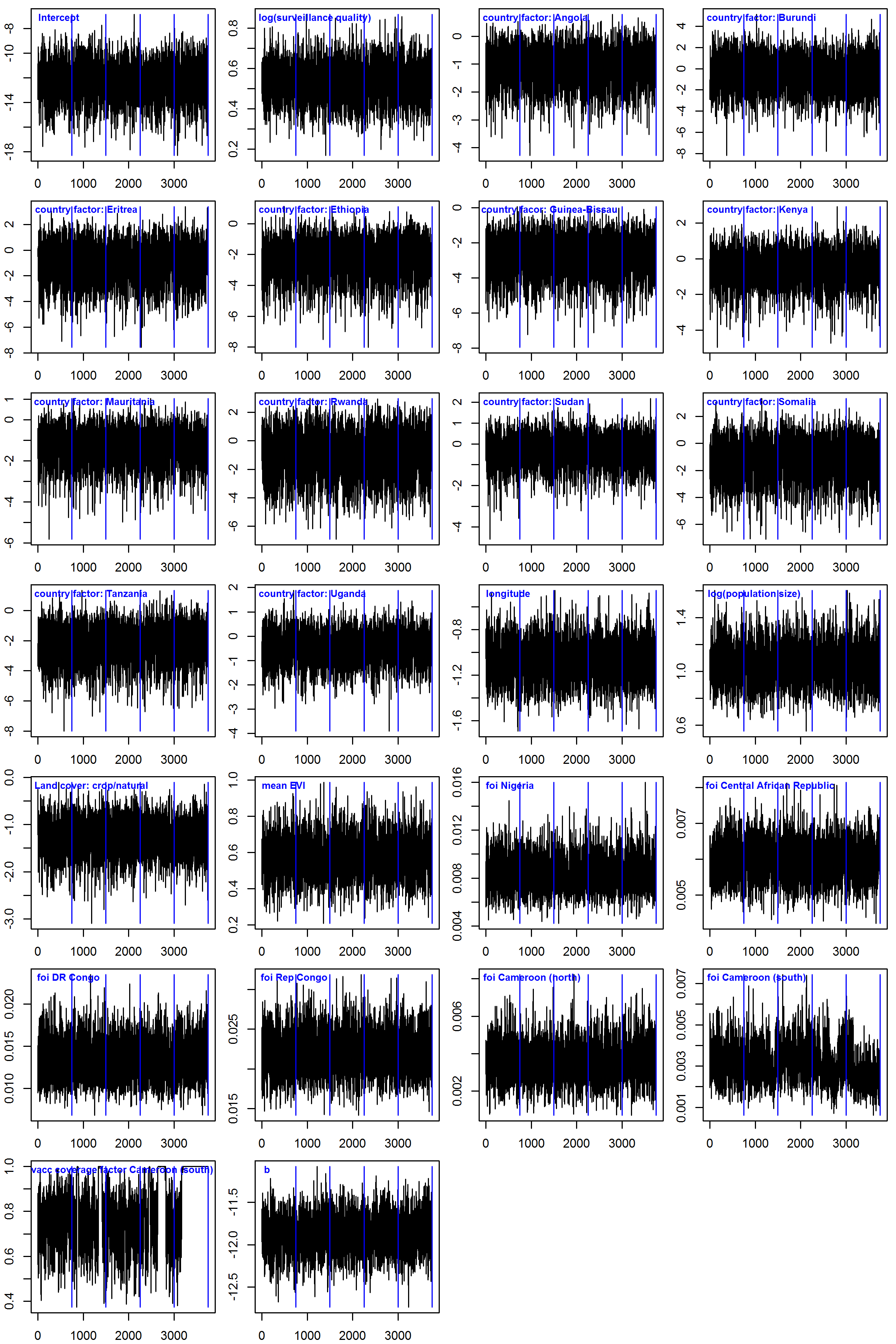

Supplement: Figure S6 — MCMC posterior trace plots of model parameter estimates for the baseline model, thinned by a factor 800. (PNG) [file pmed.1001638.s006.png]

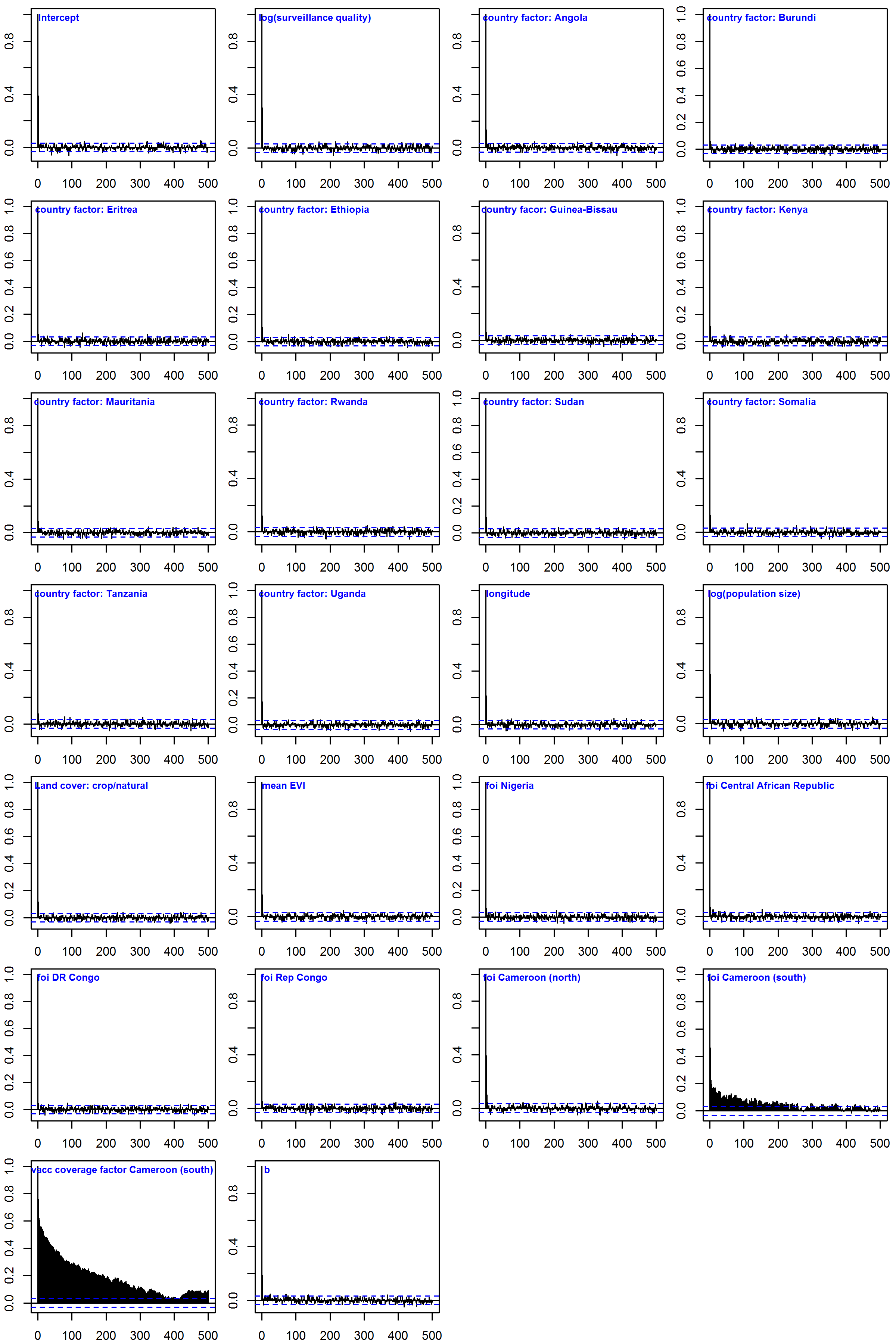

Supplement: Figure S7 — Auto-correlation in posterior estimates of the model parameters for the baseline model. Posterior MCMC samples were thinned by a factor 800. (PNG) [file pmed.1001638.s007.png]

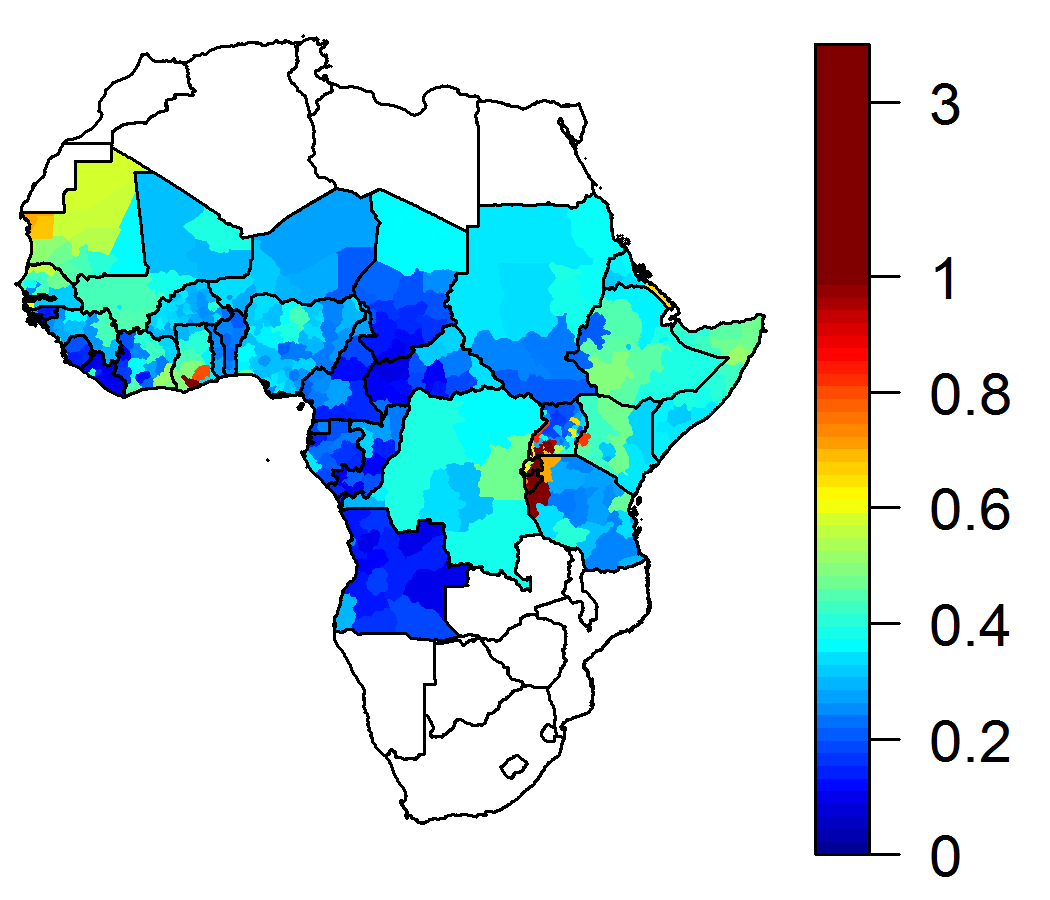

Supplement: Figure S8 — Coefficient of variation of the force of infection estimates. Countries not considered endemic for yellow fever are shown in white. (PNG) [file pmed.1001638.s008.png]
