# Supplementary material for: Yellow Fever in Africa: Estimating the Burden of Disease and Impact of Mass Vaccination from Outbreak and Serological Data
Source: PLoS Med. 2014 May 6;11(5):e1001638. doi: 10.1371/journal.pmed.1001638 (PMC4011853; doi:10.1371/journal.pmed.1001638)
Supplement: Table S1 — Coverage and year of introduction of the yellow fever vaccine into the routine Enhanced Programme of Immunization by country. (PDF) [file pmed.1001638.s009.pdf]

Yellow Fever in Africa: Estimating the burden of disease and impact of mass vaccination from outbreak and serological data

Supporting Table S1: Coverage and year of introduction of the yellow fever vaccine into the routine Enhanced Programme of Immunization by country.

Country	Year introduced	Coverage in 2011 (%)
Angola	1991	64
Benin	2002	72
Burkina Faso	1987	82
Central African Republic	1989	62
Côte d'Ivoire	1987	49
Cameroon	2004	75
Democratic Republic of the Congo	1992	48
Republic of Congo	2004	90
Gabon	1991	55
Ghana	1992	91
Guinea	2002	56
Gambia	1985	91
Guinea-Bissau	2008	61
Kenya	1992	1
Liberia	2001	39
Mali	1992	61
Niger	1988	51
Nigeria	1993	75
Senegal	1987	81
Sierra Leone	2002	80
Chad	1992	33
Togo	1992	65

Source: [1]

References

1. World Health Organization. Immunization surveillance, assessment and monitoring. http://www.who.int/immunization_monitoring/data/data_subject/en/index.html. Accessed 11 June 2012.
