# Supplementary material for: Yellow Fever in Africa: Estimating the Burden of Disease and Impact of Mass Vaccination from Outbreak and Serological Data
Source: PLoS Med. 2014 May 6;11(5):e1001638. doi: 10.1371/journal.pmed.1001638 (PMC4011853; doi:10.1371/journal.pmed.1001638)
Supplement: Table S2 — Covariates considered in the regression modeling, significance level in univariate models and cluster association. (PDF) [file pmed.1001638.s010.pdf]

Yellow Fever in Africa: Estimating the burden of disease and impact of mass vaccination from outbreak and serological data

Supporting Table S2: Covariates considered in the regression modelling, significance level in univariate models and cluster association.

varname	significance	cluster
longitude	***	1
latitude	***	2
altitude	***	3
mean night temperature	***	3
max night temperature	***	3
log(population)	***	4
LC: evergreen broadleaf forest	**	5
LC: deciduous broadleaf forest	**	6
LC: closed shrubland	***	7
LC: open shrublands	**	8
LC: woody savannas	***	9
LC: urban and built-up	.	10
LC: cropland/natural vegetation mosaic	***	11
LC: barren or sparsely vegetated	*	12
min day temperature	***	13
mean day temperature	***	13
max day temperature	**	13
min night temperature	***	14
max EVI	***	15
mean EVI	**	15
min MIR	**	16
mean MIR	.	16
max rainfall	***	17
mean rainfall	**	17
min rainfall	*	18
LC: water	-	NA
population	-	NA
LC: permanent wetlands	-	NA
LC: grasslands	-	NA
max MIR	-	NA
min EVI	-	NA
urban proportion	-	NA
LC: croplands	-	NA
LC: savannas	-	NA

significance levels *** $p<0.001$, ** $0.001 \le p<0.01$, * $0.01 \le p<0.05$, . $0.05 \le p<0.01$, - $p \ge 0.01$.
