# Supplementary material for: Yellow Fever in Africa: Estimating the Burden of Disease and Impact of Mass Vaccination from Outbreak and Serological Data
Source: PLoS Med. 2014 May 6;11(5):e1001638. doi: 10.1371/journal.pmed.1001638 (PMC4011853; doi:10.1371/journal.pmed.1001638)
Supplement: Text S1 — Demographic data analysis. (PDF) [file pmed.1001638.s011.pdf]

Yellow Fever in Africa: Estimating the burden of disease and impact of mass vaccination from outbreak and serological data

Text S1
Demographic data analysis

Tini Garske[1], Maria D Van Kerkhove[1], Sergio Yactayo[2], Olivier Ronveaux[3], Rosamund F Lewis[4], J Erin Staples[5], William Perea[2], Neil M Ferguson[1] with the YF Expert Committee*

[1] MRC Centre for Outbreak Analysis, Department of Infectious Disease Epidemiology, Imperial College London, UK

[2] World Health Organization, Geneva, Switzerland

[3] Immunization and Vaccine Development, World Health Organization, Ouagadougou, Burkina Faso

[4] Ottawa Public Health, Ottawa, Ontario, Canada

[5] Division of Vector-Borne Infectious Diseases, National Center for Zoonotic, Vector-Borne, and Enteric Diseases, Centers for Disease Control and Prevention, Fort Collins, CO, United States

*Expert Committee members: Donald Burke, Fernando De La Hoz, Bryan Grenfell, Peter M Hansen, Raymond Hutubessy, Rosamund Lewis, William Perea, Olivier Ronveaux, Erin Staples, Sergio Yactayo

In order to disaggregate the age distributions in the UN WPP dataset given in 5 year age bands we used data on the infant and child mortality rates also obtained from the UN WPP[1]. To this end, consider a cohort born at time $t-a$, which will be aged a at time t, and since birth this cohort will have experienced time and age dependent mortality rates $\mu(t,a)$. The probability of survival to time t and age a of a person in this cohort is given by

$$S(a,t)=\exp\left[-\int_0^a \mu(t-a+a^*,a^*)da^*\right]=\exp\left[-\int_{t-a}^t \mu\big(\tau,\tau-(t-a)\big)d\tau\right].$$

Assuming that mortality (and birth) rates change slowly over time, such that they can be considered approximately constant over a 5-year period, this simplifies to

$$S(a,t)=\exp\left[-\int_0^a \mu(t,a^*)da^*\right]=\exp\left[-\int_{t-a}^t \mu\big(t,\tau-(t-a)\big)d\tau\right]$$

As the dataset only gives infant and child mortality rate, the age-dependence of the mortality rate is assumed to follow a step function,

$$\mu(t,a)=\begin{cases}\mu_0(t) & a<1\\ \mu_1(t) & 1\le a<5\end{cases}$$

With this, the survival to age $a<1$ simplifies to

$$S(a,t)=\exp\left[-\int_0^a \mu_0(t)da*\right]=e^{-\mu_0(t)a}$$

whereas survival to age $1\le a<5$ simplifies to

$$S(a,t)=\exp\left[-\int_0^1 \mu_0(t)da*-\int_1^a \mu_1(t)da*\right]=e^{-\mu_0(t)}e^{-\mu_1(t)(a-1)}$$

For constant birth and death rates over the past 5 years, the number of infants (age <1) alive at time t is proportional to

$$n_{01}=\int_0^1 e^{-\mu_0(t)a}da=\frac{1-e^{-\mu_0(t)}}{\mu_0(t)}$$

and the number of children aged between 1 and <5 is proportional to

$$n_{15}=\int_1^5 e^{-\mu_0(t)}e^{-\mu_1(t)(a-1)}da=e^{-\mu_0(t)}\frac{1-e^{-4\mu_1(t)}}{\mu_1(t)}$$

The proportion of infants among children under 5 is therefore given by

$$\frac{n_{01}}{n_{01}+n_{15}}=\left[1+\frac{\mu_0 e^{-\mu_0}\left(1-e^{-4\mu_1}\right)}{\mu_1\left(1-e^{-\mu_0}\right)}\right]^{-1}.$$

The infant and child mortality rates in the data are given as deaths per 1000 live births within the first year, m_{01}, or within the first 5 years, m_{05}.

Of 1000 births, after 1 year there are $1000-m_{01}=1000e^{-\mu_0}$ infants alive, such that the mortality rate in the first year, μ_0, is given by

$$\mu_0=-\ln\left[1-\frac{m_{01}}{1000}\right]$$

In the following 4 years, the number of children alive reduces further from $1000e^{-\mu_0}$ to $1000-m_{05}$, such that $1000e^{-\mu_0}e^{-4\mu_1}=1000-m_{05}$, or

$$\mu_1=-\frac{1}{4}\left[\ln\left(1-\frac{m_{05}}{1000}\right)-\ln\left(1-\frac{m_{01}}{1000}\right)\right]=-\frac{1}{4}\left[\ln\left(1-\frac{m_{05}}{1000}\right)+\mu_0\right]$$

The UN WPP dataset provides estimates of the infant mortality for the whole period from 1950 to 2100, whereas child mortality rate is estimated only from 1995 onwards. Both infant and child mortality rates decreased steadily over time and the proportion of infants does not vary substantially between 1995 and 2100. For the times prior to 1995, the infant and child mortality estimates from 1995 were used to generate the annual age cohorts.

As vaccination against yellow fever started shortly after the development of the yellow fever vaccine around 1940 population sizes for the 1940s were also needed, but these years are not covered in the WPP. Population growth in the 1950s was slow, so here it was assumed that the populations stayed effectively constant throughout the 1940s at the level estimated for 1950.

References

1. United Nations, Department of Economic and Social Affairs, Population Division, Population Estimates and Projections Section. World Population Prospects: The 2010 Revision. http://esa.un.org/wpp/Excel-Data/population.htm. Accessed 11 Jun 2011.
