# Supplementary material for: Yellow Fever in Africa: Estimating the Burden of Disease and Impact of Mass Vaccination from Outbreak and Serological Data
Source: PLoS Med. 2014 May 6;11(5):e1001638. doi: 10.1371/journal.pmed.1001638 (PMC4011853; doi:10.1371/journal.pmed.1001638)
Supplement: Text S2 — Sensitivity analysis: impact of the covariates included. (PDF) [file pmed.1001638.s012.pdf]

Yellow Fever in Africa: Estimating the burden of disease and impact of mass vaccination from outbreak and serological data

Text S2
Sensitivity analysis: Impact of the covariates included

Tini Garske[1], Maria D Van Kerkhove[1], Sergio Yactayo[2], Olivier Ronveaux[3], Rosamund F Lewis[4], J Erin Staples[5], William Perea[2], Neil M Ferguson[1] with the YF Expert Committee*

[1] MRC Centre for Outbreak Analysis, Department of Infectious Disease Epidemiology, Imperial College London, UK

[2] World Health Organization, Geneva, Switzerland

[3] Immunization and Vaccine Development, World Health Organization, Ouagadougou, Burkina Faso

[4] Ottawa Public Health, Ottawa, Ontario, Canada

[5] Division of Vector-Borne Infectious Diseases, National Center for Zoonotic, Vector-Borne, and Enteric Diseases, Centers for Disease Control and Prevention, Fort Collins, CO, United States

*Expert Committee members: Donald Burke, Fernando De La Hoz, Bryan Grenfell, Peter M Hansen, Raymond Hutubessy, Rosamund Lewis, William Perea, Olivier Ronveaux, Erin Staples, Sergio Yactayo

As described in the main paper, 15 regression models with differing included covariates were investigated in the full model framework in the present study. All 15 models provided a good fit to the data with differences in the fit between the models being non-significant, in the sense that the uncertainty in the goodness of fit criteria (BIC and AUC) within any one model was larger than the differences between models. In this section, we present the results from the 15 models using the default standard deviation $\sigma = 2$ for the prior distributions on the country factors. The effect of using different prior standard deviations is demonstrated in the next section.

The number of parameters fitted in the regression models was substantial, varying from 24 to 26 in the different models. Some of these parameters were fitted in all models, whereas others were only included in some models. The parameters that were part of the regression models included an intercept, environmental covariates as well as factors determining the surveillance quality for countries not participating in the YFSD. Of these, the exact environmental covariates included varied between models, with the intercept and country factors being fitted in all models. Furthermore, in the full model framework parameters were fitted to serological survey data: a force of infection parameter for each survey considered, as well as one additional parameter describing how much the vaccination coverage was reduced in the rural study population in southern Cameroon[1] compared to the level expected from the vaccination coverage dataset.

Although both components of the model were fitted using a combined likelihood, the regression and serosurvey likelihoods appeared to be fairly independent from one another, resulting in parameter estimates for the serological parameters that were highly consistent between the different regression models (Panels C and E in Figure S2.1). Owing to the inclusion of different parameters in the regression component of the model, the estimates for these parameters varied more between models, although they too were very consistent (Panels A, B and D in Figure S2.1).

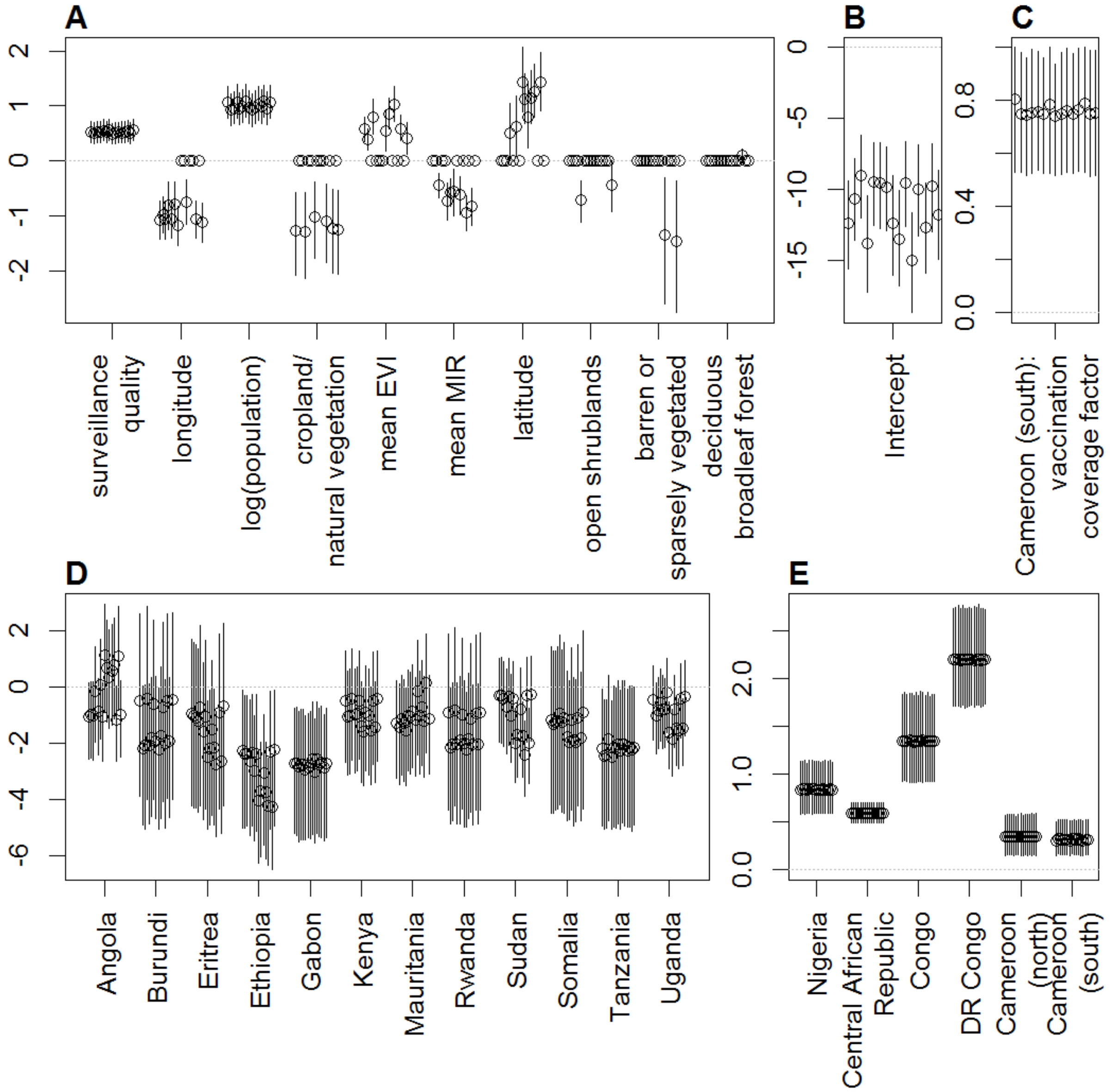

Figure S2.1: Parameter estimates from the different models. A) environmental parameters included in any of the 15 best fitting regression models, B) intercept in the regression models, C) factor adjusting the level of the vaccination coverage in the analysis of the serological survey in southern Cameroon [1], D) country factors fitted to estimate the surveillance quality for countries not participating in the YFSD, E) force of infection estimates from the serological surveys. For each parameter, the point estimates (circles) and 95%CIs (lines) are shown for all 15 models. Parameters not included in a model are indicated at zero value (panel A only).

The model predictions obtained with the parameter estimates were then used to estimate the detection probabilities in all countries and the force of infection for all provinces. Generally the correlations between the detection probabilities and the forces of infection estimated for the

different models were high, although it was evident that the models fell into two groups: (a) models 1-7, 13 and 15, and (b) models 8-12 and 14 (Figure S2.2). Interestingly, the models in the former group all included longitude as a covariate, whereas the models in the latter group all included latitude as a covariate. Of the three models including both longitude and latitude, two (models 4 and 6) were included in the longitude group, and one (model 10) was included in the latitude group.

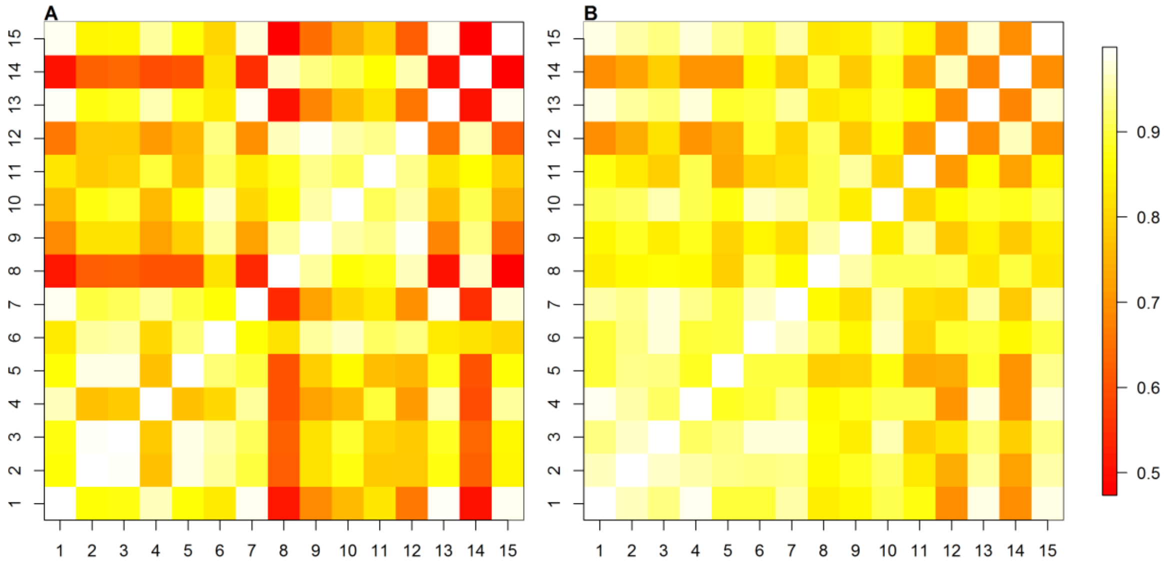

Figure S2.2: Correlation matrices of the detection probabilities (A) and forces of infection (B) estimated for the different models 1 to 15.

The burden estimates were highly consistent across all 15 models (Figure S2.3). Although the models including latitude as a covariate tended to have slightly lower burden estimates, the differences were much smaller than the overall uncertainty associated with the estimates.

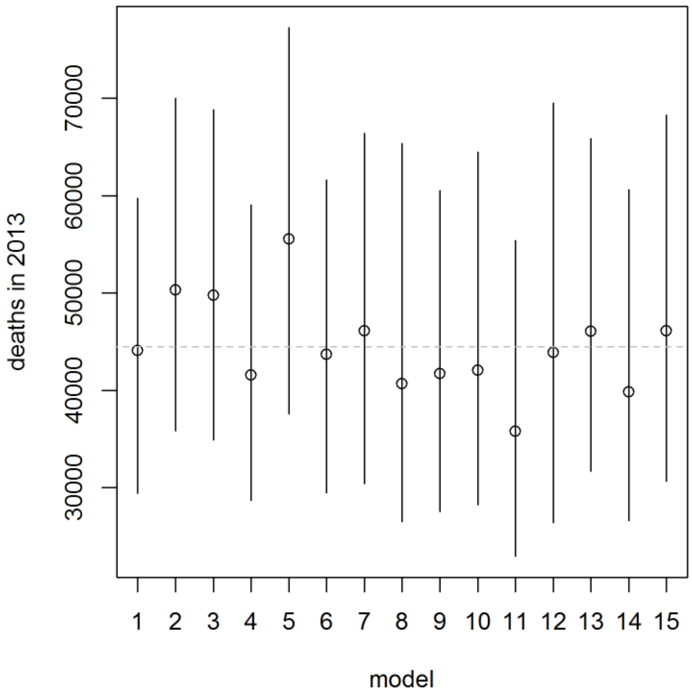

Figure S2.3: Burden in terms of the number of deaths estimated with 95% CI for 2013 for the different models. The dashed horizontal line shows the mean across all models.

References

1. Kuniholm MH, Wolfe ND, Huang CY-H, Mpoudi-Ngole E, Tamoufe U, et al. (2006) Seroprevalence and Distribution of Flaviviridae, Togaviridae, and Bunyaviridae Arboviral Infections in Rural Cameroonian Adults. The American Journal of Tropical Medicine and Hygiene 74: 1078-1083.
