# Supplementary material for: Yellow Fever in Africa: Estimating the Burden of Disease and Impact of Mass Vaccination from Outbreak and Serological Data
Source: PLoS Med. 2014 May 6;11(5):e1001638. doi: 10.1371/journal.pmed.1001638 (PMC4011853; doi:10.1371/journal.pmed.1001638)
Supplement: Text S3 — Sensitivity analysis: impact of the standard deviation of the prior distribution on the country factors. (PDF) [file pmed.1001638.s013.pdf]

Yellow Fever in Africa: Estimating the burden of disease and impact of mass vaccination from outbreak and serological data

Text S3
Sensitivity analysis: Impact of the standard deviation of the prior distribution on the country factors

Tini Garske[1], Maria D Van Kerkhove[1], Sergio Yactayo[2], Olivier Ronveaux[3], Rosamund F Lewis[4], J Erin Staples[5], William Perea[2], Neil M Ferguson[1] with the YF Expert Committee*

[1] MRC Centre for Outbreak Analysis, Department of Infectious Disease Epidemiology, Imperial College London, UK

[2] World Health Organization, Geneva, Switzerland

[3] Immunization and Vaccine Development, World Health Organization, Ouagadougou, Burkina Faso

[4] Ottawa Public Health, Ottawa, Ontario, Canada

[5] Division of Vector-Borne Infectious Diseases, National Center for Zoonotic, Vector-Borne, and Enteric Diseases, Centers for Disease Control and Prevention, Fort Collins, CO, United States

*Expert Committee members: Donald Burke, Fernando De La Hoz, Bryan Grenfell, Peter M Hansen, Raymond Hutubessy, Rosamund Lewis, William Perea, Olivier Ronveaux, Erin Staples, Sergio Yactayo

The impact of the prior distribution for country factors on model results is demonstrated using model 1 only, but these results are representative of the general behaviour of all models.

While the estimated magnitude of country factors was strongly dependent on the standard deviation assumed for their prior distribution, there was only a low dependence of the other regression parameters on the standard deviation of the prior (Figure S3.1). The estimates of parameters used to fit the serological data showed no dependence (data not shown). As expected, factors for those countries for which there were no yellow fever reports in the dataset showed the strongest dependence on the assumed prior distributions. However, the estimates for those countries where there were both provinces with and without yellow fever reports were much less sensitive to the prior distributions, as these estimates were limited by the proportion of provinces reporting yellow fever within the country.

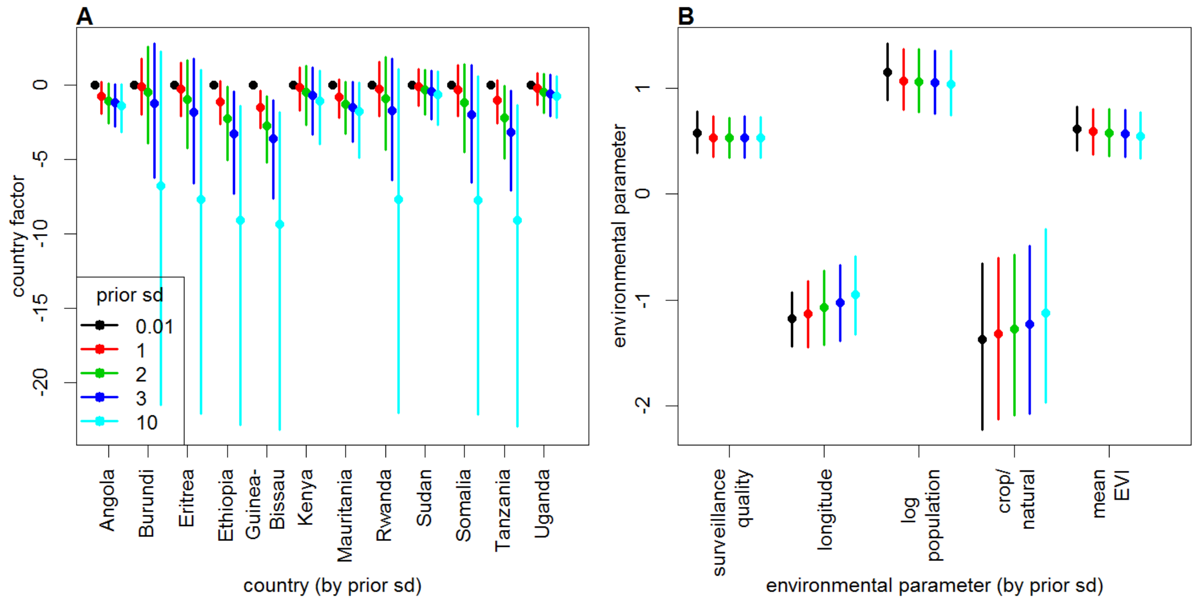

Figure S3.1: Dependence of the regression parameter estimates on the prior standard deviation σ.

For low values of the standard deviation on the country factor prior, the detection probabilities for the countries with fitted country factors were on the low end of the spectrum given by the countries for which surveillance quality data was used to estimate the detection probability. As the standard deviation was increased, the detection probabilities separated into two groups, one containing the countries with surveillance quality data and those countries with fitted country factors in which some yellow fever events were reported, the other consisting of the countries with neither yellow fever reports nor data on surveillance quality (Figure S3.2). While it is sensible to assume that the countries that do not participate in the YFSD (and therefore had no surveillance quality data) had lower detection probabilities than those that do, the differences between the countries seen for the higher values of the standard deviation on the country factor prior were probably not realistic. Therefore a value of $\sigma = 2$ was chosen as a baseline as this allowed some variation of the detection probabilities between countries but did not generate unrealistic differences in detection probabilities between countries.

Interestingly, the burden estimates were only weakly dependent on the assumed prior distribution on country factors (Figure S3.3). In East Africa the burden estimates increased with increasing standard deviation due to the lower estimates of the detection probability in these countries, this was compensated by decreasing burden estimates for West Africa due to the slight dependence of the other model parameters on the standard deviation, most notably the longitude. However, these trends were small compared to the overall uncertainty in the burden estimates.

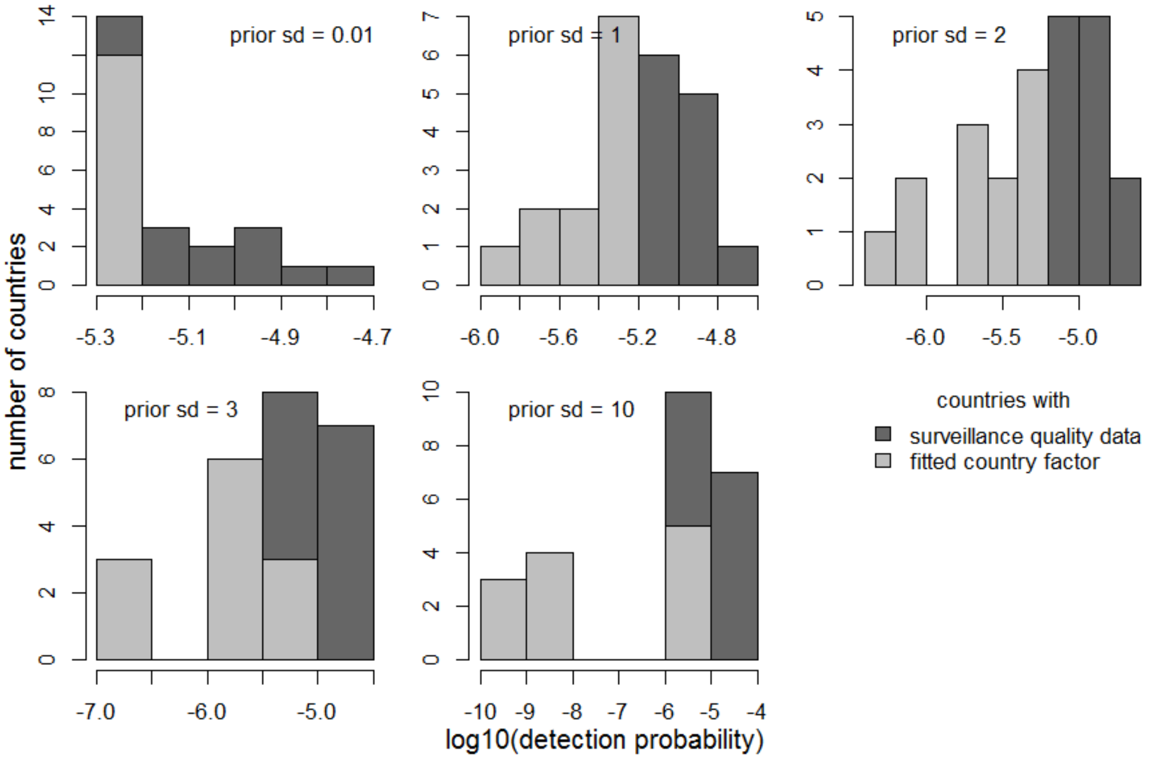

Figure S3.2: Histograms of the detection probabilities for each country by standard deviation of the prior distributions.

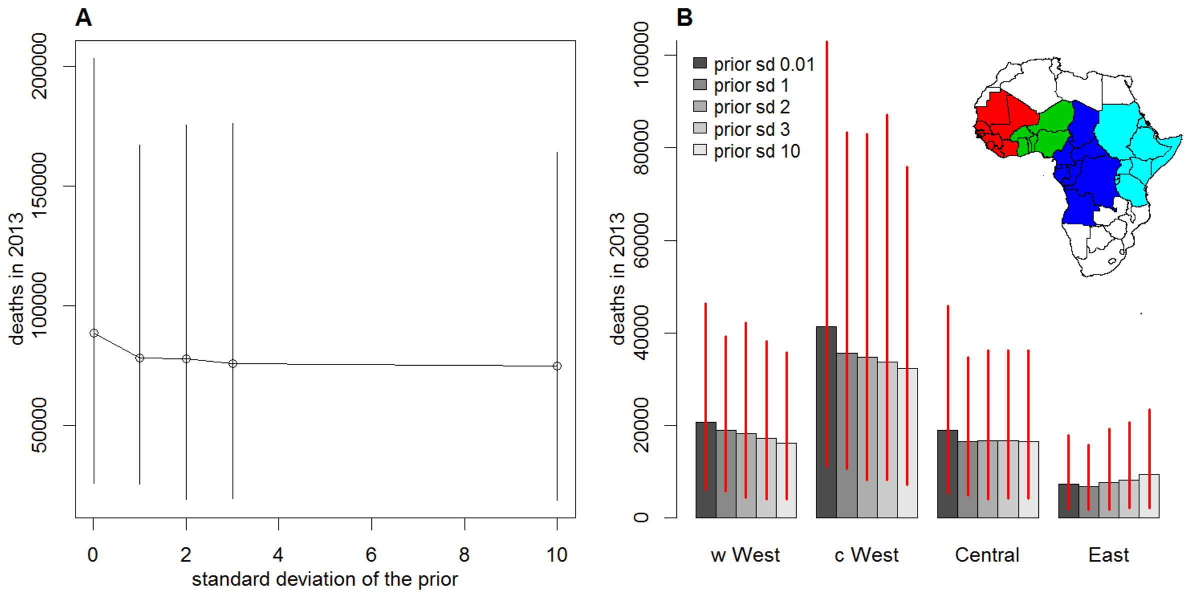

Figure S3.3: Burden of yellow fever by prior standard deviation as described by the estimated number of deaths in 2013. A) total burden across Africa, B) burden by region. Inset map shows the regions: red = western West Africa, green = central West, blue = Central Africa and turquoise = East Africa. Circles (left) and bars (right) indicate point estimates, vertical lines indicate 95% CIs.
