# Supplementary material for: Yellow Fever in Africa: Estimating the Burden of Disease and Impact of Mass Vaccination from Outbreak and Serological Data
Source: PLoS Med. 2014 May 6;11(5):e1001638. doi: 10.1371/journal.pmed.1001638 (PMC4011853; doi:10.1371/journal.pmed.1001638)
Supplement: Text S4 — Sensitivity analysis: impact of alternative vaccination coverage scenarios. (PDF) [file pmed.1001638.s014.pdf]

Yellow Fever in Africa: Estimating the burden of disease and impact of mass vaccination from outbreak and serological data

Text S4
Sensitivity analysis: Impact of alternative vaccination coverage scenarios

Tini Garske[1], Maria D Van Kerkhove[1], Sergio Yactayo[2], Olivier Ronveaux[3], Rosamund F Lewis[4], J Erin Staples[5], William Perea[2], Neil M Ferguson[1] with the YF Expert Committee*

[1] MRC Centre for Outbreak Analysis, Department of Infectious Disease Epidemiology, Imperial College London, UK

[2] World Health Organization, Geneva, Switzerland

[3] Immunization and Vaccine Development, World Health Organization, Ouagadougou, Burkina Faso

[4] Ottawa Public Health, Ottawa, Ontario, Canada

[5] Division of Vector-Borne Infectious Diseases, National Center for Zoonotic, Vector-Borne, and Enteric Diseases, Centers for Disease Control and Prevention, Fort Collins, CO, United States

*Expert Committee members: Donald Burke, Fernando De La Hoz, Bryan Grenfell, Peter M Hansen, Raymond Hutubessy, Rosamund Lewis, William Perea, Olivier Ronveaux, Erin Staples, Sergio Yactayo

Our estimated values of yellow fever vaccination coverage levels across Africa over time are of course subject to substantial uncertainties that are difficult to quantify. In order to provide some sensitivity analyses to some of the assumptions made in compiling this dataset, five alternative vaccination coverage scenarios were generated.

Vaccine efficacy 90%

While the efficacy of the yellow fever vaccine is thought to be extremely high causing sero-conversion in 99% of recipients within 30 days[1,2], which we have approximated assuming 100% efficacy for the main results. However, we also investigated a scenario assuming a lower efficacy of 90% to allow for vaccine failures due to reasons such as inappropriate storage or administration of the vaccine, or lower immunogenicity in immunocompromised sub-populations such as HIV positives.

Non-random vaccine allocation

While for the baseline scenario it was assumed that vaccine would be allocated randomly in subsequent vaccination campaigns targeting the same population (i.e. the chances of being vaccinated in the second campaign would not depend on the previous vaccination status), in this

scenario the assumption was made that access to vaccination was distributed highly unevenly across the population, such that in subsequent campaigns in each age group first all previously vaccinated individuals would get a second dose before any previously unvaccinated individuals would be vaccinated, mimicking the situation that access to health care interventions is likely to be better in urban than remote areas. This scenario results in somewhat lower vaccination coverage in areas where there have been several subsequent vaccination campaigns, particularly if they took place over a short period of time. In practice, this had the biggest impact for the historic mass vaccination campaigns resulting in a lower coverage in the older population today.

Alternative population size

Estimates of population sizes in Africa can vary substantially between datasets, and as for many vaccination campaigns (particularly the historic mass vaccination campaigns and reactive campaigns) the available information was the number of doses administered into a population rather than the coverage achieved, different assumptions about the underlying population size would lead to a different vaccination coverage. Therefore scenarios were created using a population size 25% smaller or larger than the baseline assumption, resulting higher or lower vaccination coverage, respectively.

Alternative historic mass vaccination campaigns

Owing to the substantial time lapse since these campaigns, the data available was rather coarse, giving the number of doses administered by year or decade across all African countries targeted, with mass vaccination campaigns typically implemented every four years[3,4]. Two data sources were identified giving different numbers of doses used. The more detailed dataset[5] was used for the baseline scenario, whereas the alternative dataset by Moreau et al[6] quoted rather higher numbers of vaccine doses used, and these numbers were used to create this alternative scenario.

Using these alternative vaccination coverage scenarios did not have a significant effect on the estimated disease burden from yellow fever, although the point estimates of the burden for the reduced vaccine efficacy and the non-random vaccine allocation are slightly elevated compared to the baseline scenario as in both these scenarios the effective vaccination coverage is somewhat lower (see Table S4.1).

Table S4.1: Estimated deaths for 2013 caused by yellow fever for the different vaccination coverage scenarios, illustrated with the results for model 1 using a prior standard deviation of $\sigma = 2$.

Scenario	Deaths 2013
Baseline	78000 (19000 -- 180000)
Vaccine efficacy 90%	86000 (24000 -- 190000)
Non-random vaccine allocation	84000 (22000 -- 190000)
High coverage	77000 (21000 -- 180000)
Low coverage	77000 (20000 -- 170000)
Alternative historic vaccination campaigns	78000 (22000 -- 180000)

References

1. Monath TP (2001) Yellow fever: an update. The Lancet Infectious Diseases 1: 11-20.
2. World Health Organization (2013) Vaccines and vaccination against yellow fever. WHO Position Paper - June 2013. Weekly Epidemiological Record 88: 269-284.
3. World Health Organization (1990) Yellow Fever in 1988. Weekly Epidemiological Record 65: 213-219.
4. Vainio J, Cutts F (1998) Yellow Fever. World Health Organization. WHO/EPI/GEN/98.11.
5. Durieux C (1956) Mass yellow fever vaccination in French Africa south of the Sahara. Monograph Series World Health Organization pp. 115-122.
6. Moreau JP, Girault G, Dram I, Perraut R (1999) [Reemergence of yellow fever in West Africa: lessons from the past, advocacy for a control program]. Bulletin de la Société de pathologie exotique 92: 333-336.
