# Supplementary material for: Yellow Fever in Africa: Estimating the Burden of Disease and Impact of Mass Vaccination from Outbreak and Serological Data
Source: PLoS Med. 2014 May 6;11(5):e1001638. doi: 10.1371/journal.pmed.1001638 (PMC4011853; doi:10.1371/journal.pmed.1001638)
Supplement: Text S5 — Sensitivity Analysis: alternative model structures. (PDF) [file pmed.1001638.s015.pdf]

Yellow Fever in Africa: Estimating the burden of disease and impact of mass vaccination from outbreak and serological data

Text S5
Sensitivity analysis: Alternative model structures

Tini Garske[1], Maria D Van Kerkhove[1], Sergio Yactayo[2], Olivier Ronveaux[3], Rosamund F Lewis[4], J Erin Staples[5], William Perea[2], Neil M Ferguson[1] with the YF Expert Committee*

[1] MRC Centre for Outbreak Analysis, Department of Infectious Disease Epidemiology, Imperial College London, UK

[2] World Health Organization, Geneva, Switzerland

[3] Immunization and Vaccine Development, World Health Organization, Ouagadougou, Burkina Faso

[4] Ottawa Public Health, Ottawa, Ontario, Canada

[5] Division of Vector-Borne Infectious Diseases, National Center for Zoonotic, Vector-Borne, and Enteric Diseases, Centers for Disease Control and Prevention, Fort Collins, CO, United States

*Expert Committee members: Donald Burke, Fernando De La Hoz, Bryan Grenfell, Peter M Hansen, Raymond Hutubessy, Rosamund Lewis, William Perea, Olivier Ronveaux, Erin Staples, Sergio Yactayo

Introduction

The model for estimating the burden of yellow fever consisted of several components. In order to limit the overall complexity, some simplifying assumptions and choices regarding the model structure were made that could be refined. Here two alternative model structures are investigated to assess the sensitivity to some of these assumptions.

Model definitions

Variable infection detection probability

In the basic model presented in the main paper, an inherent assumption was that the detection probability per infection, θ_c, varies between countries, but is constant throughout the period of observation from 1987 to 2011. However, it could be argued that with the set-up of the YFSD in 2005, surveillance should have been improved in the countries participating in the database. In order to model this effect, we partitioned the yellow fever presence/absence dataset by province into two, one covering 1987 to 2004, and the second covering 2005 to 2011. This doubles the size of the dataset the regression model was fitted to. As the periods covered in the eras pre and post

implementation of the YFSD differed (18 vs. 7 years, respectively), the probability of a report would be expected to differ substantially even in the absence of any changes of the detection probability. Therefore, an additional intercept β_0 was included in the model predictions for the early period, such that the model predictions given in equation (1) in the main paper become

$$q_{\text{pre}} = 1 - \exp\left[-e^{\beta_0 + X\beta}\right], \tag{S5.1}$$

$$q_{\text{post}} = 1 - \exp\left[-e^{X\beta}\right], \tag{S5.2}$$

and the log-likelihood for the generalised linear model component (equation (2) in the main text) is

$$\ln L_{\text{glm}} = \sum_i \left[y_{\text{pre},i} \ln\left(q_{\text{pre},i}\right) + \left(1 - y_{\text{pre},i}\right) \ln\left(1 - q_{\text{pre},i}\right) + y_{\text{post},i} \ln\left(q_{\text{post},i}\right) + \left(1 - y_{\text{post},i}\right) \ln\left(1 - q_{\text{post},i}\right) \right], \tag{S5.3}$$

where $y_{\text{pre},i}$ and $y_{\text{post},i}$ described the presence or absence of yellow fever reports in province i in the pre- or post-YFSD eras, respectively. The model predictions were connected to the transmission intensity by allowing for different detection probabilities pre and post for the countries participating in the YFSD, whereas the detection probabilities for non-participating countries (fitted as country factors) were kept constant. In order to achieve this, the parameter b anchoring the overall transmission intensity was replaced by two parameters b_{pre} and $b_{\text{post}} = b_{\text{pre}} + b_{SD}$, where b_{SD} described the increase in detection probability due to the YFSD and was set to 0 for non-participating countries. Equation (6) in the main text was therefore replaced by

$$\ln n_{\text{pre},i} = \beta_0 + X\beta - \beta_c - b_{\text{pre}} \quad \text{and} \quad \ln\left(-\ln\left(1 - \theta_{\text{pre},c}\right)\right) = \beta_c + b_{\text{pre}}, \tag{S5.4}$$

$$\ln n_{\text{post},i} = X\beta - \beta_c - b_{\text{post}} \quad \text{and} \quad \ln\left(-\ln\left(1 - \theta_{\text{post},c}\right)\right) = \beta_c + b_{\text{post}}. \tag{S5.5}$$

The fitting of the force of infection to the serological survey data, obtaining estimates for the parameters b_{pre} and b_{SD}, the detection probabilities $\theta_{\text{pre/post},c}$ and finally the force of infection across the epidemic region was achieved analogously to the basic model presented in the main text.

Fitting to annual yellow fever reports

In both the basic model and the model with variable detection probability, the dataset fitted to was the presence or absence of yellow fever reports aggregated over a number of years. We further generalised the variable detection probability model by sub-dividing the reporting periods into annual periods. As in the previous model, the detection probability was assumed to change only in the countries participating in the YFSD at the introduction of that database, and stay constant throughout the pre and post introduction eras. Due to this assumption, the dataset fitted to could be combined into two sub-sets, one for the pre and one for the post era, respectively, with $y_{\text{pre/post},i}$ now indicating the number of years in the pre or post era during which yellow fever was reported in province i, instead of simply giving the presence or absence of reports during that era. In this model, the model predictions were interpreted as the annual probability of a yellow fever report, which only changed between the pre and post era for those countries participating in the YFSD.

Hence instead of fitting an additional intercept for one era and then calculating the resulting change in detection probability via the parameter b_{SD}, the change in detection probability could be directly fitted with a parameter β_{SD} that was only used in the post era, and set to zero for non-participating countries. With this, the model predictions were given as

$$q_{\mathrm{pre}} = 1 - \exp\left[-e^{X\beta}\right], \tag{S5.6}$$

$$q_{\mathrm{post}} = 1 - \exp\left[-e^{X\beta+\beta_{\mathrm{SD}}}\right]. \tag{S5.7}$$

Splitting these up into the transmission and detection parts yielded

$$\ln\left(n_{\mathrm{annual},i}\right) = X\beta - \beta_c - b \tag{S5.8}$$

for both pre and post eras, where $n_{\mathrm{annual},i}$ is the average annual number of infections required to explain the data, and

$$\ln\left(-\ln\left(1-\theta_{\mathrm{pre},c}\right)\right) = \beta_c + b, \tag{S5.9}$$

$$\ln\left(-\ln\left(1-\theta_{\mathrm{post},c}\right)\right) = \beta_c + \beta_{\mathrm{SD}} + b, \tag{S5.10}$$

where equations (S5.8) to (S5.10) replaced equation (6) from the main paper.

Results

In the following, the baseline model presented in the main text is referred to as the single-level model (as it assumed a constant detection probability across all years), whereas the model fitted to the presence or absence of yellow fever reports for the two periods from 1987 to 2004 and 2005 to 2011 shall be referred to as the two-level presence model, as detection probabilities were allowed to vary between the two periods considered. The model fitted to the number of years during each of the two periods for which yellow fever was reported in any province shall be referred to as the two-level annual model.

The alternative model structures were evaluated using the same 15 regression models in terms of the covariates included as those used for the baseline model presented in the main paper. For space reasons, we only present results for regression model 1 with $\sigma = 2$ for the country factor priors. However, these results are representative of the results obtained from the other models and assumptions on σ.

On the whole, the fitted parameters for the alternative models took very similar values to those fitted in the single-level model. The dependence of the parameters on the covariates included and the prior standard deviation was also very similar, though there were some differences in the values of the country factors. These directly impacted the detection probability and force of infection estimates, and therefore also the burden estimates as shown below.

Despite the post YFSD period only covering 7 years compared to the 18 years covered in the early period, yellow fever was reported in more provinces in the recent period, and the model predictions mirror this increase in reports between the early and recent periods (Figure S5.1).

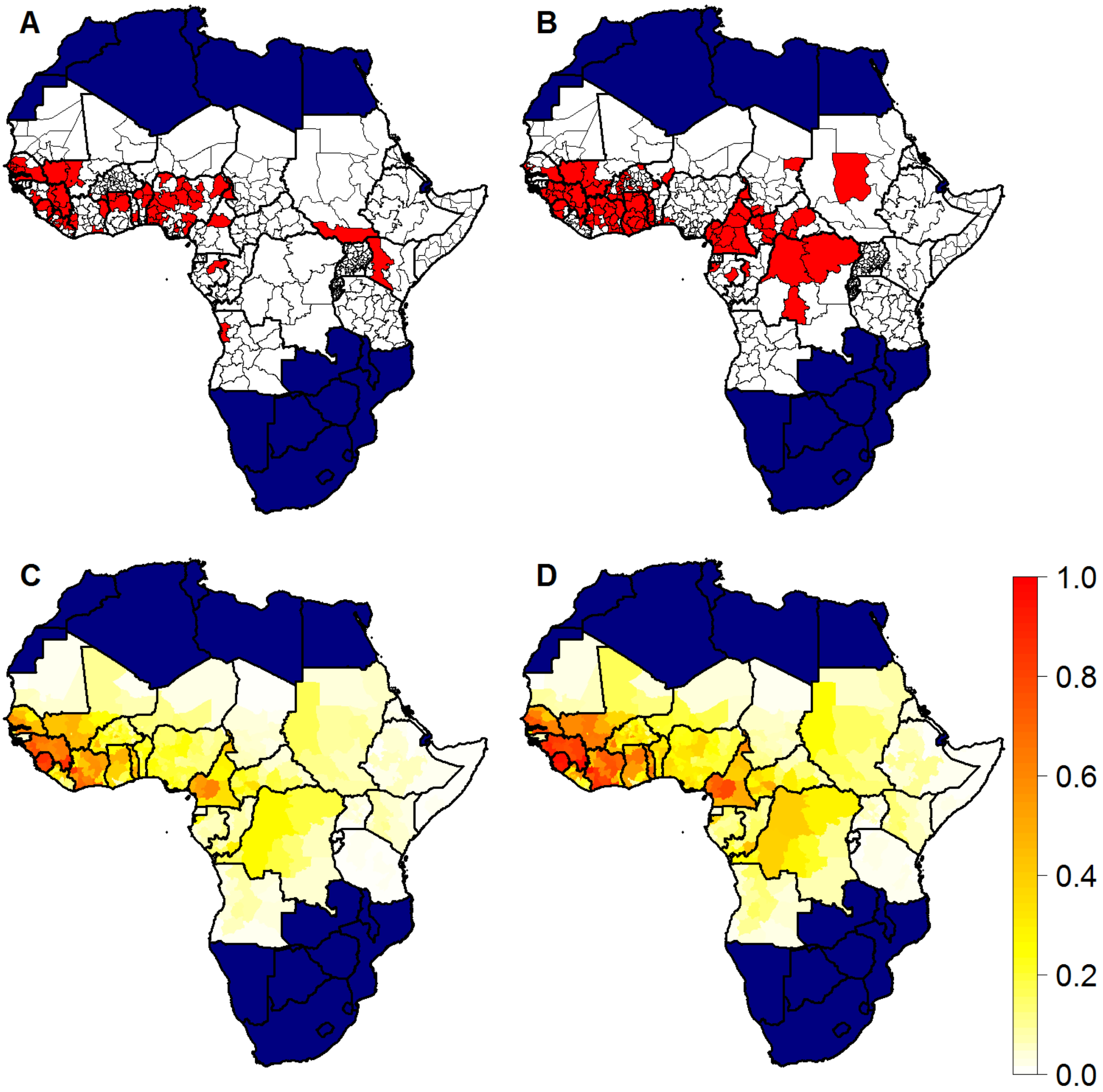

Figure S5.1: A and B: dataset the two-level presence model was fitted to, indicating the presence (red) or absence (white) of yellow fever reports during the periods from 1987 to 2004 (A) and from 2005 to 2011 (B), C and D: model predictions from the two-level presence model for the periods from 1987 to 2004 (C) and 2005 to 2011 (D), indicating the probability of at least one yellow fever report during the period considered.

The dataset for the two-level annual model which recorded the number of years during which yellow fever was reported for each period also showed an increase in reports between the pre- and post-YFSD periods, although the exceptional situation in Nigeria became evident, as the large amplification of yellow fever transmission observed there around 1990 gave rise to reports across many provinces over several years (Panels A and B in Figure S5.2). In contrast to the two-level presence model, here the model predictions were interpreted as the annual probability of a yellow fever report, and a priori would therefore not expected to differ between the early and recent periods if the rate of yellow fever reports found in the data stayed the same. Due to the model

structure, the model predictions for countries not participating in the YFSD stayed constant across both periods, whereas those in participating countries increased, following the patterns seen in the data (Panels C and D in Figure S5.2).

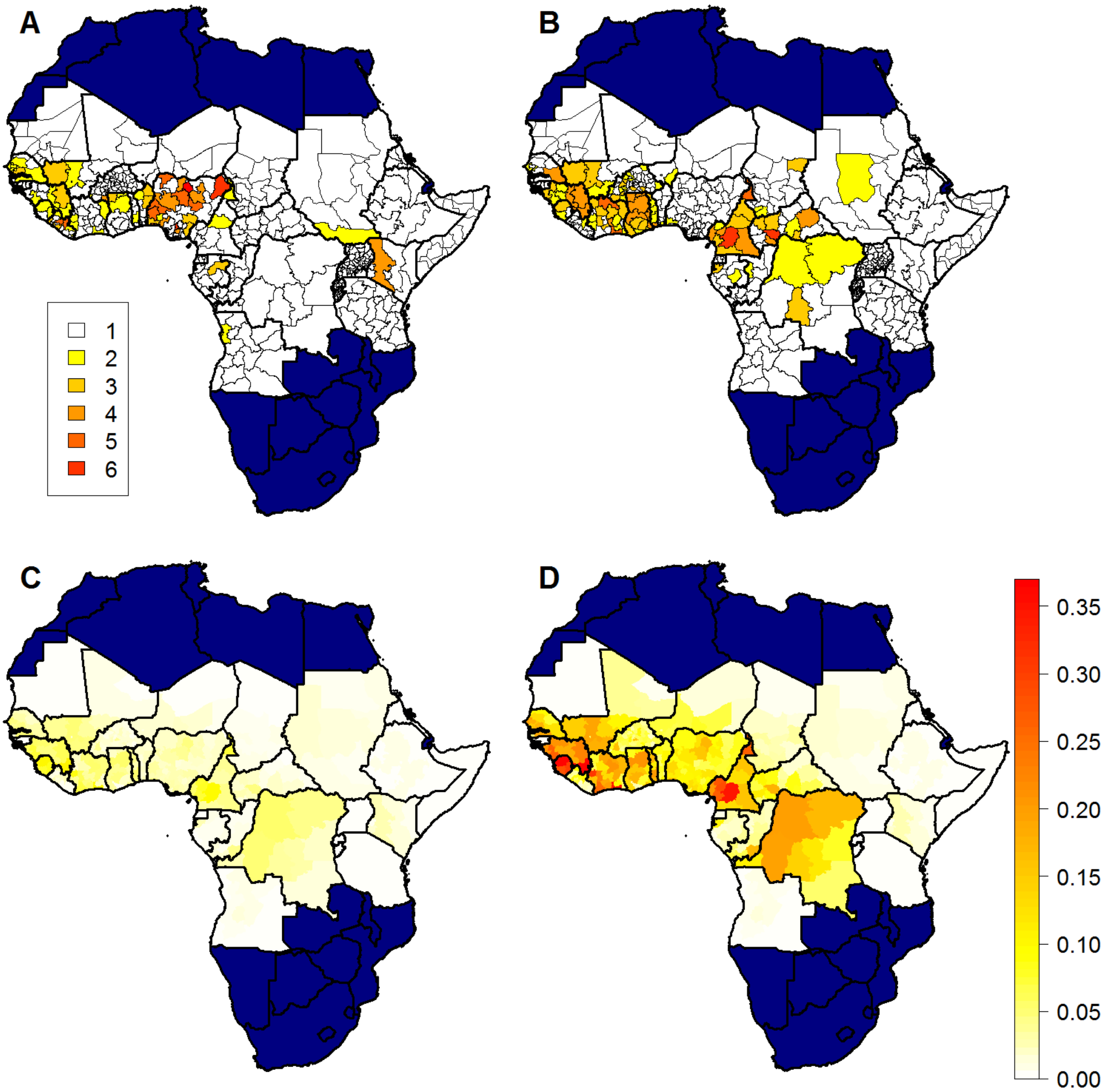

Figure S5.2: A and B: dataset the two-level annual model was fitted to, indicating the number of years during which yellow fever was reported for the periods from 1987 to 2004 (A) and from 2005 to 2011 (B), C and D: model predictions from the two-level annual model for the periods from 1987 to 2004 (C) and 2005 to 2011 (D), indicating the annual probability of a yellow fever report.

The increase in yellow fever reports between the early and recent periods could be explained by increased transmission intensity, decreased vaccination coverage or increased detection probability. The models implicitly assumed that the force of infection remained constant throughout, and the recent period saw substantial investment into vaccination campaigns that raised the vaccination coverage throughout this period substantially, particularly in West Africa (Figure S3); this increase was taken into account in the model fitting. Hence the model reproduces the increase in reports between the pre- and post-YFSD periods via increases in infection detection probabilities, particularly for countries participating in the YFSD (Figure S5.3):

For the two-level presence model, the infection detection probability was estimated to have increased by a factor 3.5 (95%CI 2.5-4.9) following the introduction of the YFSD. For the two-level annual model, the increase was slightly higher at 4.1 (95% CI 3.3 – 5.3). In the single-level baseline model, the detection probabilities were assumed to be constant over time, so one would expect them to show a moderate level that is higher than the two-level estimates for the pre-YFSD period, but lower than the 2-level estimates for the post-YFSD period in the countries participating in the YFSD, as can be seen in Figure S5.3. The two-level annual model generally resulted in higher estimates of the detection probability for countries participating in the YFSD than the two-level presence model, but comparable estimates for countries not participating (Figure S5.3, 2nd and 3rd column and row).

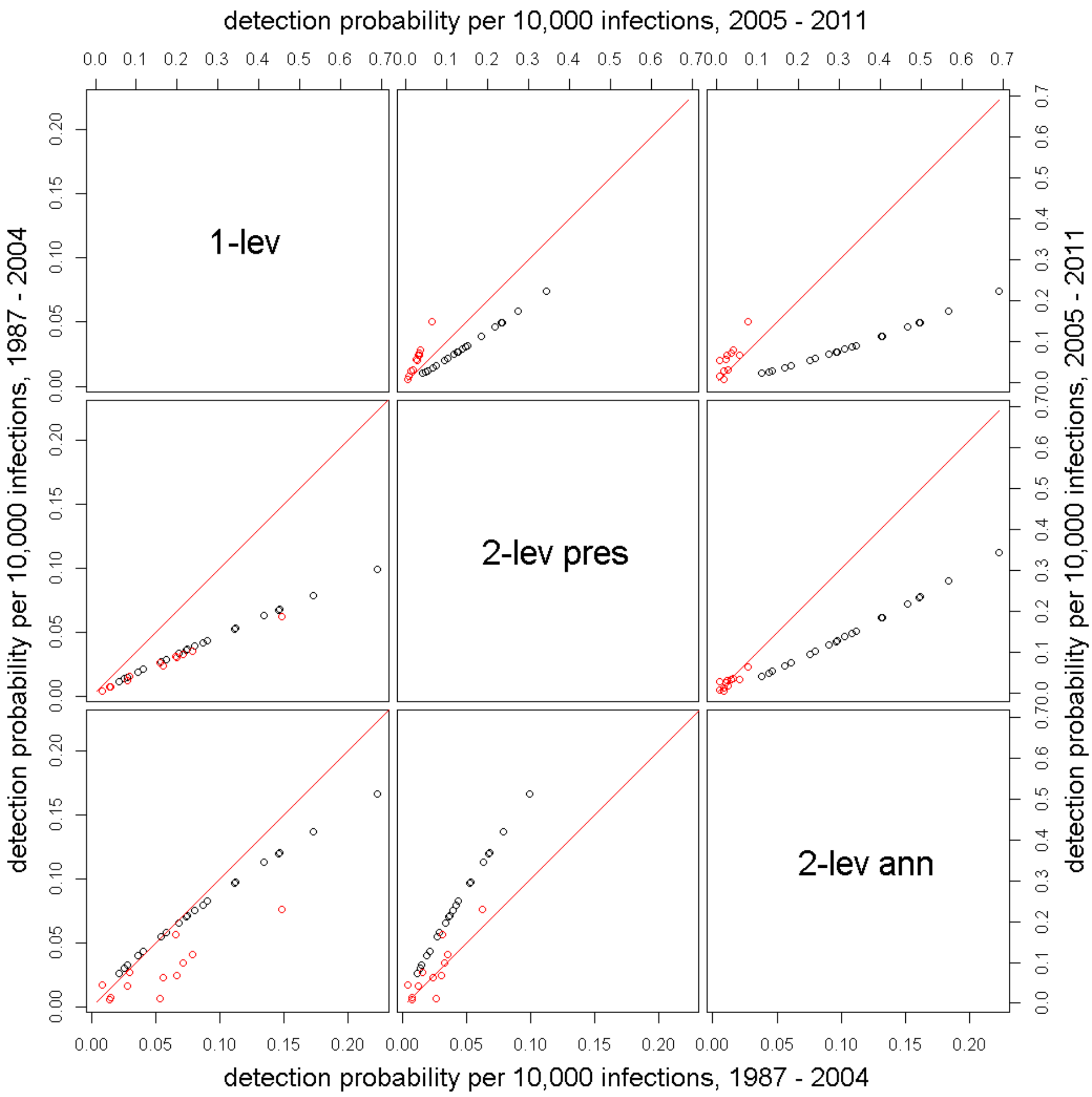

Figure S5.3: Scatter plots of the detection probabilities prior (plots below the diagonal) and post (plots above the diagonal) the introduction of the YFSD. 1st column and row (1-lev): baseline model (with a constant detection probability throughout), 2nd column and row (2-lev pres): two-level presence model, 3rd column and row (2-lev ann): two-level annual model. Black circles: countries participating in the YFSD, red circles: countries not participating in the YFSD. Red lines indicate unity.

The force of infection estimates were very similar between the baseline and the two-level presence model, particularly for the countries participating in the YFSD, whereas the estimates for the non-participating countries were slightly higher in the two-level presence than the baseline model. The two-level annual model on the other hand provided lower estimates of the force of infection than either the baseline or the two-level presence model, particularly for provinces with estimates of the force of infection at the higher end of the scale. Conversely, the estimates at the lower end of the scale tended to be slightly higher in the two-level annual than in the baseline model (Figure S5.4).

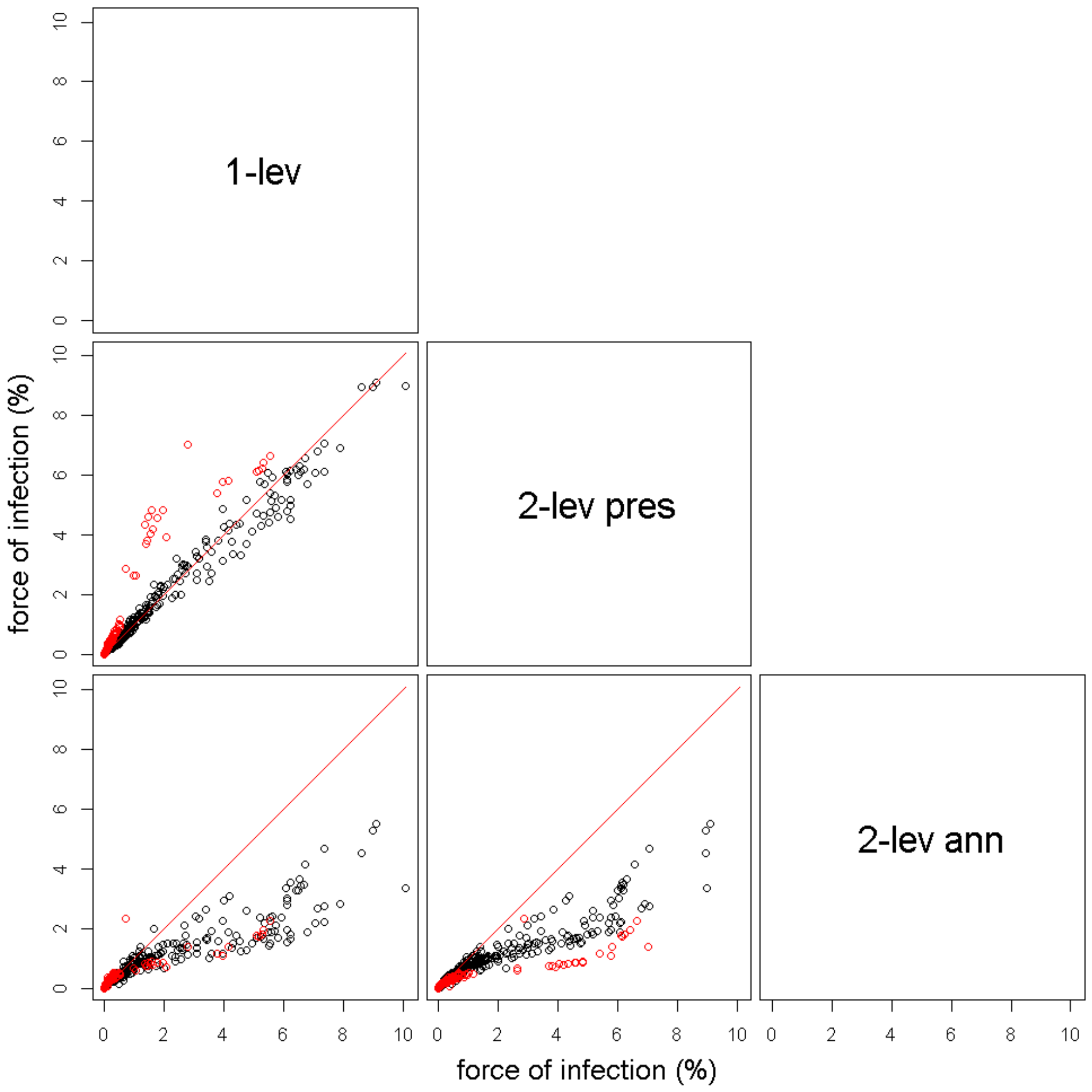

Figure S5.4: Scatter plots of the force of infection. Legend as for Figure S5.3.

The differences in force of infection estimates translated directly to differences in the burden estimates, though these were not major (Figure S5.5). The increase in burden between 1995 and 2005 seen in all model versions was largely due to population growth. In this period there were no large scale mass vaccination campaigns that would have impacted on vaccination coverage significantly. Routine infant immunization was performed in several countries, but the mostly modest coverage achieved (Table S1) was not sufficient overall to counteract the effects of

population growth. The reduction in burden seen between 2005 and 2013 can mostly be attributed to the GAVI funded mass vaccination campaigns, which managed to reduce the absolute number of deaths despite the effects of population growth. However the impact of these mass vaccination campaigns was estimated to be slightly lower in the two-level annual model, as for that model the force of infection estimates were slightly lower than obtained from the single-level model in the high risk areas in west Africa where the mass vaccination campaigns were performed.

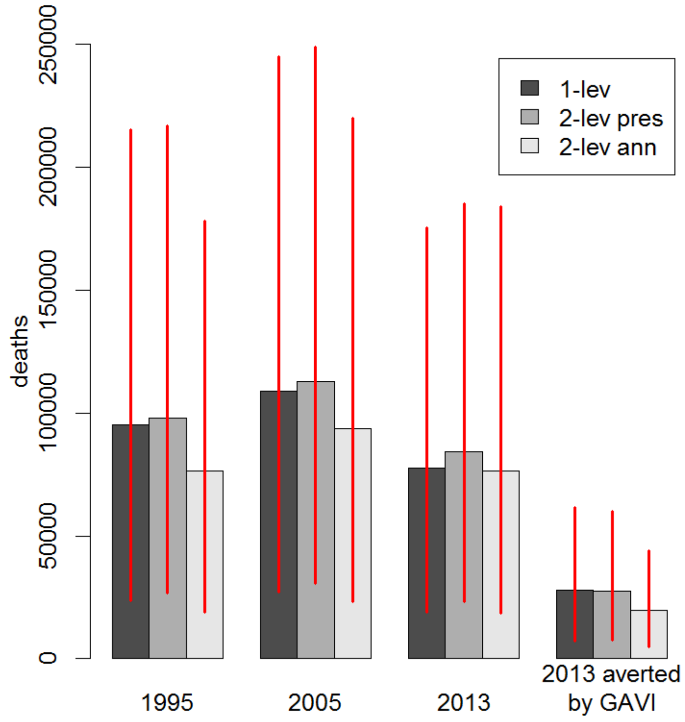

Figure S5.5: Burden in terms of the estimated number of deaths for the different model structures evaluated for various years, as well as the number of deaths averted by the GAVI-funded mass-vaccination campaigns implemented between 2006 and 2012. Grey bars indicate point estimates, red lines indicate 95% CIs.
